# Inteligência Artificial Aplicada à Avaliação de Risco Cardiovascular Baseada em Eletrocardiograma: Uma Revisão Sistemática

**DOI:** 10.36660/abc.20250790

**Published:** 2026-07-22

**Authors:** Maria Clara Mantoan Pinheiro, Lívia Felberg, Isadora Cristine Reis Sguizzato Bozzi, Antônio Luiz Pinho Ribeiro, Gabriela Miana de Mattos Paixão

**Affiliations:** 1 Universidade Federal de Minas Gerais Belo Horizonte MG Brasil Universidade Federal de Minas Gerais, Belo Horizonte, MG – Brasil

**Keywords:** Inteligência Artificial, Fatores de Risco de Doenças Cardíacas, Eletrocardiografia

## Abstract

As doenças cardiovasculares são a principal causa de mortalidade no mundo, destacando a necessidade de estratégias eficazes de predição de risco e detecção precoce. Embora o eletrocardiograma (ECG) seja um exame amplamente disponível e de baixo custo, sua interpretação tradicional é limitada pela subjetividade. A inteligência artificial (IA) surgiu como uma abordagem promissora, capaz de extrair informações prognósticas ocultas dos sinais de ECG. Esta revisão sistemática teve como objetivo avaliar estudos originais que aplicaram técnicas de IA a ECGs para predição de risco cardiovascular e mortalidade. Foram incluídos estudos originais que utilizaram sinais de ECG como única variável de entrada para modelos de IA, com foco em desfechos de risco cardiovascular. Uma busca sistemática foi realizada em diferentes bases de dados, e os dados fora msintetizados de forma narrativa. Onze estudos foram incluídos, predominantemente coortes retrospectivas que aplicaram redes neurais convolucionais (CNNs) para prever risco cardiovascular ou mortalidade. As amostras eram majoritariamente compostas por populações adultas de países de alta renda. Os desfechos primários incluíram mortalidade por todas as causas, morte cardiovascular e eventos cardiovasculares adversos maiores (MACE). Os valores de AUROC variaram de 0,63 a 0,961 nos conjuntos de treinamento, com alguns modelos superando escores tradicionais de risco. Os modelos de IA‑ECG demonstraram potencial para detectar doença subclínica, permitindo estratificação precoce de risco mesmo em ECGs normais. No entanto, persistem desafios relacionados à diversidade populacional, interpretabilidade dos modelos e validação prospectiva. A aplicação de IA à análise de ECG representa um avanço promissor na avaliação personalizada do risco cardiovascular. Contudo, mais pesquisas são necessárias para garantir a segurança, a eficácia e a integração clínica equitativa dessas tecnologias.

## Graphical abstract

**Figura Central:**
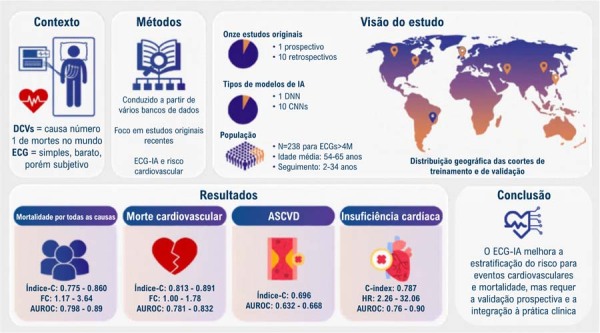
: Inteligência Artificial Aplicada à Avaliação de Risco Cardiovascular Baseada em Eletrocardiograma: Uma Revisão Sistemática

## Introdução

As doenças cardiovasculares (DCVs) são a principal causa de mortalidade no mundo, englobando tanto condições transmissíveis quanto não transmissíveis.^1^ Portanto, a estratificação do risco cardiovascular e a detecção precoce dessas doenças são essenciais. Nesse contexto, o eletrocardiograma (ECG) destaca-se como uma ferramenta bem estabelecida, devido à sua ampla disponibilidade, baixo custo e simplicidade operacional. No entanto, a interpretação do ECG apresenta uma limitação importante: sua subjetividade, uma vez que depende de critérios visuais que variam conforme o examinador.

Nos últimos anos, a inteligência artificial (IA) ganhou destaque significativo, especialmente na área da medicina, abrindo novas fronteiras na análise de dados. Abordagens baseadas em *machine learning* (ML) e *deep learning* (DL) demonstraram capacidade de reconhecer padrões complexos e sutis que podem passar despercebidos por observadores humanos. Isso levou à hipótese de que integrar IA à análise de ECG (IA-ECG) pode permitir a extração de informações prognósticas e de estratificação de risco cardiovascular anteriormente não identificadas.^2,3^

Os modelos atuais de estimativa de risco cardiovascular apresentam limitações importantes, pois nem sempre refletem com precisão a realidade individual do paciente e, muitas vezes, não incorporam dados do ECG. O uso de métodos adicionais, como o escore de cálcio coronariano, apresenta bom desempenho na reestratificação do risco cardiovascular; entretanto, o custo permanece uma barreira significativa, especialmente em países de baixa e média renda.^4^ Como resultado, um número considerável de pacientes permanece desassistido, sobretudo aqueles de populações diversas cujas características sociais e raciais não são adequadamente consideradas. Isso reforça a necessidade de validação em populações heterogêneas – uma prática ainda ausente em alguns dos estratificadores de risco existentes.^4^

Embora a maioria dos eventos ateroscleróticos possa ser prevenida por meio de estratégias de promoção da saúde e do controle de fatores de risco conhecidos, variáveis individuais persistem, incluindo baixa adesão ao tratamento e desigualdade social.^2^ Nesse contexto, a aplicação de IA à análise de ECG pode oferecer uma alternativa para apoiar o desenvolvimento de modelos de predição mais sensíveis, personalizados e integrados.^3^

O objetivo deste artigo é fornecer uma revisão sistemática atualizada e crítica das principais aplicações de IA na interpretação de ECG, com foco na predição de eventos cardiovasculares adversos maiores (MACE). O artigo examina os avanços recentes, os desafios associados à implementação em larga escala e as perspectivas para sua adoção na prática clínica. Os principais achados desta revisão sistemática estão resumidos na Ilustração Central.

## Métodos

Esta revisão sistemática foi conduzida de acordo com as diretrizes do *Preferred Reporting Items for Systematic Reviews and Meta-Analyses* (PRISMA).^5^ O protocolo foi registrado no PROSPERO (1039916).

A metodologia desta revisão sistemática seguiu o modelo “PICOTS” (*Population, Intervention, Comparator, Outcome, Timing,* e *Study design*). Foram incluídos estudos envolvendo populações adultas que aplicaram modelos de IA utilizando exclusivamente sinais de ECG como entrada, sem a necessidade de um grupo comparador explícito. Os desfechos primários foram mortalidade por todas as causas, morte cardiovascular, insuficiência cardíaca (IC), infarto do miocárdio (IAM), acidente vascular cerebral (AVC) e doença cardiovascular aterosclerótica (DCVA). Os períodos de seguimento variaram entre os estudos, com predominância de delineamentos de coorte retrospectiva.

### Critérios de elegibilidade

Incluímos artigos de pesquisa original publicados em inglês nos últimos cinco anos que avaliaram diretamente o uso de IA – especificamente redes neurais convolucionais (CNNs, do inglês *convolutional neural networks*) ou redes neurais profundas (DNNs, do inglês *deep neural networks*) – aplicadas exclusivamente a ECGs para prever risco cardiovascular em populações adultas (≥18 anos). Os estudos elegíveis avaliaram o risco ou a predição de mortalidade por todas as causas, DCVA e eventos cardiovasculares adversos maiores (MACE), incluindo IAM, IC, AVC e morte cardiovascular.

Foram excluídos estudos que: (1) integraram dados de ECG com outras informações clínicas, laboratoriais ou de imagem; (2) focaram exclusivamente no diagnóstico de arritmias; (3) foram restritos a populações específicas de pacientes; ou (4) eram revisões narrativas, relatos de caso ou não disponibilizavam o texto completo.

### Fontes de informação e estratégia de busca

A busca na literatura foi realizada nas bases PubMed, Cochrane Library, Scopus e ScienceDirect. As buscas foram limitadas a artigos publicados entre 2020 e 2025, e a última busca foi realizada em maio de 2025. Os seguintes termos em inglês e seus sinônimos foram utilizados como descritores: *“Artificial Intelligence”, “Machine Learning”, “Deep Learning”, “Electrocardiography”, “Electrocardiogram”, “ECG”, “Cardiovascular Risk Factors”, “Risk Assessment”, “Cardiovascular Risk”, “Heart Disease Risk”*. Os operadores booleanos “AND” e “OR” foram aplicados para combinar os termos.

### Processo de seleção

O processo de seleção consistiu em quatro etapas: identificação, triagem, avaliação de elegibilidade e inclusão. A busca inicial identificou 1118 registros, dos quais 1056 foram excluídos após a triagem de títulos por não abordarem o foco central da revisão. Dos 62 registros restantes, 45 foram excluídos após a avaliação dos resumos e remoção de duplicatas. Em seguida, seis registros adicionais foram excluídos após a leitura do texto completo por não atenderem aos critérios de inclusão. Por fim, onze estudos cumpriram todos os critérios de elegibilidade e foram incluídos na revisão.

**Figura 1:**
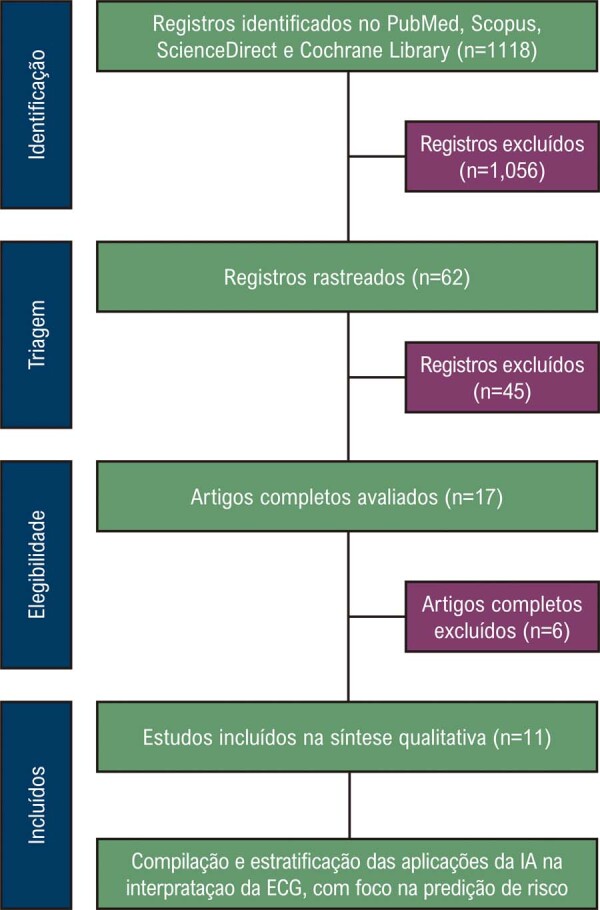
– Diagrama de fluxo PRISMA: Processo de seleção de estudos

Dois revisores conduziram independentemente todas as fases do processo de seleção. Quaisquer discrepâncias foram resolvidas por meio de discussão com um terceiro revisor. Todo o fluxo de seleção está apresentado no diagrama PRISMA (Figura 1).

### Processo de coleta de dados e itens de dados

A extração de dados foi realizada de forma independente por dois revisores, utilizando formulários padronizados criados no Microsoft Excel. Os dados extraídos incluíram o delineamento do estudo, características da população, tipo de modelo de IA (CNN ou DNN), detalhes da aquisição do ECG e os desfechos cardiovasculares avaliados. Variáveis adicionais coletadas incluíram o cenário do estudo, tamanho da amostra, os procedimentos de treinamento e validação dos modelos de IA e se houve validação externa.

## Resultados

Um total de onze estudos originais foi incluído nesta revisão sistemática. Suas características estão resumidas na Tabela 1. Dez estudos eram coortes retrospectivas^6-15^e um estudo tinha delineamento prospectivo.^16^ Os estudos analisaram uma ampla variedade de sinais de ECG, predominantemente ECGs de 12 derivações, com alguns também avaliando registros de derivação única (Derivação I).^6,7^

**Tabela 1:** – Características dos estudos de inteligência artificial aplicada na eletrocardiografia (IA-ECG) incluídos

Autores	Ano de publicação	Título do artigo	Tipo do estudo	Técnicas de IA	Tipo de sinal de input	População	Número de ECGs	Idade média (anos)	Homens (%)	Seguimento (média de anos)	Validação externa	População de validação
Sau et al.^6^	2024	Artificial intelligence-enabled electrocardiogram for mortality and cardiovascular risk estimation: a model development and validation study	Retrospectivo	CNN	ECG de 12 derivações e de derivação única (DI)	BIDMC (EUA)	1 163 401	NR	NR	5.46	Sim	CODE (Brasil) SaMi-Trop (Brasil) ELSA-Brazil (Brasil) UK Biobank (Reino Unido)
Hughes et al.^7^	2023	A deep learning-based electrocardiogram risk score for long term cardiovascular death and disease	Retrospectivo	CNN	ECG de 12 derivações e de derivação única (DI)	Stanford Health Care (EUA)	500 000	59	54%	5	Sim	Cedars-Sinai Medical Center (EUA) Columbia University Irving Medical Center (EUA)
Al-Alusi et al.^8^	2025	A deep learning digital biomarker to detect hypertension and stratify cardiovascular risk from the electrocardiogram	Retrospectivo	CNN	ECG de 12 derivações	Massachusetts General Hospital (EUA)	752 415	57,3 ± 16,8	51,6%	5.6–7.8	Sim	Brigham and Women’s Hospital (EUA)
Sau et al.^9^	2025	Artificial intelligence-enhanced electrocardiography for the identification of a sex-related cardiovascular risk continuum: a retrospective cohort study	Retrospectivo	CNN	ECG de 12 derivações	BIDMC (EUA)	1 163 401	57,7 ± 18,69	47,9%	3.4–4.8	Sim	UK Biobank (Reino Unido)
Gao et al.^16^	2025	Electrocardiograph analysis for risk assessment of heart failure with preserved ejection fraction: A deep learning model	Retrospectivo	CNN	ECG de 12 derivações	First Hospital of Hebei Medical University (China)	238	NR	50,8%	2	Não	Validação interna prospectiva
Raghunath et al.^10^	2020	Prediction of mortality from 12-lead electrocardiogram voltage data using a deep neural network	Retrospectivo	CNN	ECG de 12 derivações	Geisinger (USA)	1 169 662	58 ± 18	47%	34	Não	Validação interna cruzada 5-Fold
Dhingra et al.^11^	2025	Heart failure risk stratification using artificial intelligence applied to electrocardiogram images: a multinational study	Retrospectivo	CNN	ECG de 12 derivações	YNHHS (USA)	286 880	57	43,4%	10	Sim	UK Biobank (Reino Unido) ELSA-Brazil (Brasil)
Lin et al.^12^	2025	A multitask deep learning model utilizing electrocardiograms for major cardiovascular adverse events prediction	Retrospectivo	CNN	ECG de 12 derivações	CGMH (Taiwan)	2 821 889	60 ± 14,5	50,2%	12	Sim	TSGH (Taiwan)
Butler et al.^13^	2023	A generalizable electrocardiogram-based artificial intelligence model for 10-year heart failure risk prediction	Retrospectivo	CNN	ECG de 12 derivações	ARIC (EUA)	14 613	54,2	44,7%	10	Sim	MESA (USA)
Sun et al.^14^	2023	Towards artificial intelligence-based learning health system for population-level mortality prediction using electrocardiograms	Retrospectivo	DL	ECG de 12 derivações	Alberta (Canadá)	1 605 268	65,80 ± 17,25	56,81%	13	Não	NR
Lin et al.^15^	2025	ECG-surv: A deep learning-based model to predict time to 1-year mortality from 12-lead electrocardiogram	Retrospectivo	CNN	ECG de 12 derivações	CGMH (Taiwan)	4 932 573	60,5 ± 16,8	52,5%	12	Sim	Tri-Service General Hospital (Taiwan)

IA: Inteligência Artificial; ARIC: Atherosclerosis Risk in Communities Study (Estudo de Risco de Aterosclerose em Comunidades); BIDMC: Beth Israel Deaconess Medical Center; BWH: Brigham and Women’s Hospital; CGMH: Chang Gung Memorial Hospital; CODE: Clinical Outcomes in Digital Electrocardiology; DL: Deep Learning (Aprendizado Profundo); DNN: Deep Neural Network (Rede Neural Profunda); ECG: Eletrocardiograma; ELSA-Brasil: Estudo Longitudinal de Saúde do Adulto; HECIUH: Health Examination Center at Inha University Hospital; HHMU: Hospital da Universidade Médica de Hebei; MESA: Multi-Ethnic Study of Atherosclerosis (Estudo Multiétnico de Aterosclerose); MGH: Massachusetts General Hospital; NR: Não reportado; SaMi-Trop: coorte brasileira de pacientes com doença de Chagas; TSGH: Tri-Service General Hospital; UK Biobank: Biobanco do Reino Unido; YNHHS: Yale New Haven Health System.

Entre os estudos incluídos, a maioria (90,9%) empregou CNNs como a principal arquitetura para análise dos sinais de ECG.^6-13,15,16^ Especificamente, as CNNs foram utilizadas isoladamente ou em combinação com outros métodos, como camadas *Long Short-Term Memory* (LSTM), *Residual Networks* (ResNet) ou *Variational Autoencoders* (VAEs). Um estudo utilizou abordagens mais amplas de DL sem empregar explicitamente arquiteturas CNN, enquadrando-se na categoria de DNNs ou DL geral.^14^

Os tamanhos amostrais variaram substancialmente, de 238 pacientes a mais de 4 milhões de ECGs. As populações dos estudos consistiam principalmente de pacientes adultos provenientes de sistemas hospitalares associados a grandes biobancos, com idades médias geralmente variando entre 54 e 65 anos. No entanto, alguns estudos não forneceram informações demográficas detalhadas.^6^ Quanto ao período de seguimento, o mais longo relatado foi de 34 anos,^10^ enquanto o mais curto foi de dois anos.^16^

**Figura 2:**
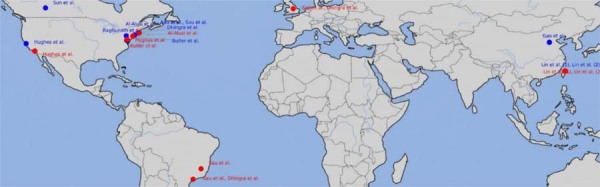
– Distribuição geográfica das coortes do estudo de IA-ECG. O mapa destaca a localização dos conjuntos de dados usados para treinamento (azul) e validação externa (vermelho) dos modelos de IA-ECG. A maioria das coortes de treinamento originou-se dos EUA, enquanto os conjuntos de dados de validação concentraram-se no Reino Unido, Brasil, Taiwan e EUA.

Como mostrado na Figura 2, a maioria das coortes utilizadas para treinar os modelos de IA era baseada nos Estados Unidos da América (EUA).^6-12^ Dois estudos utilizaram populações de Taiwan,^13,17^ enquanto um estudo incluiu populações da China^10^ e outro do Canadá.^14^

Os estudos que empregaram validação externa também incluíram participantes de países de alta renda, como o Reino Unido^6,9,11^ e os EUA.^7,8,13^ Apenas dois estudos utilizaram uma coorte do Brasil para validação externa,^6,12^ e dois estudos incluíram populações de Taiwan.^12,15^

**Figura 3:**
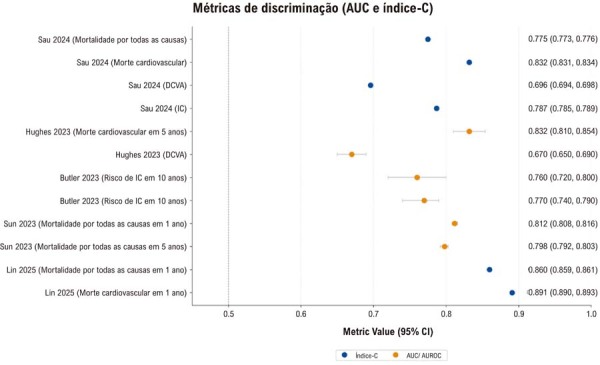
– Métricas de discriminação (AUC e índice C) para modelos de IA-ECG. Gráfico resumindo o desempenho preditivo de modelos de IA baseados em ECG em diferentes estudos e horizontes temporais. Os marcadores azuis representam o índice C (índice de concordância de Harrell) e os marcadores laranja representam a área sob a curva ROC (AUC/AUROC). Os dados são apresentados como estimativas pontuais com intervalos de confiança de 95% (IC 95%). A linha vertical tracejada em 0,5 indica o limiar para predição não discriminatória (aleatória).

**Figura 4:**
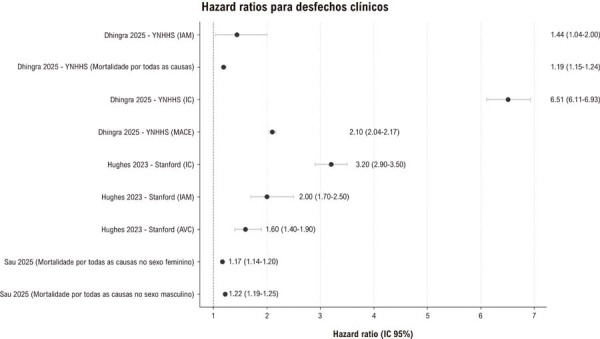
– Razões de risco para desfechos clínicos associados a modelos de IA-ECG. Gráfico mostrando a associação entre categorias de risco derivadas de IA e eventos clínicos (mortalidade, insuficiência cardíaca, eventos cardiovasculares adversos maiores, etc.). Os pontos representam as razões de risco (HR) e as barras horizontais indicam os intervalos de confiança de 95% (IC 95%). A linha vertical tracejada em 1,0 representa a hipótese nula (nenhuma diferença no risco).

Todos os desfechos relevantes para esta revisão estão apresentados na Tabela 2. Além disso, as Figuras 3 e 4 exibem *forest plots* que resumem o desempenho preditivo dos modelos de IA aplicados ao ECG nos estudos incluídos. A Figura 3 apresenta métricas de discriminação, incluindo o índice de concordância (índice-C) e a área sob a curva ROC (AUC/AUROC), enquanto a Figura 4 mostra medidas de efeito expressas como *hazard ratios* (HR) para diversos desfechos cardiovasculares, incluindo intervalos de confiança de 95% quando disponíveis.

**Tabela 2:** – Resumo do desempenho da inteligência artificial aplicada na eletrocardiografia (IA-ECG) nos diferentes desfechos clínicos

Autores	Mortalidade por todas as causas	Morte cardiovascular	DCVA	Insuficiência cardíaca	ICFEp	IAM	AVC	MACE
Sau et al.^6^	Índice-C 0,775 (0,773–0,776) Taxa de evento: NR	Índice-C 0,832 (0,831–0,834) Taxa de evento: NR	Índice-C 0,696 (0,694–0,698) Modelo SEER índice-C 0,547 (0,527–0,567) Taxa de evento: NR	Índice-C 0,787 (0,785–0,789) Taxa de evento: NR	-	-	-	-
Hughes et al.^7^	-	Stanford (12-derivações) AUC 0,832 (0,810–0,854), índice-C 0,815 (0,798–0,826); Cedars-Sinai (12-derivações) AUC 0,781 (0,767–0,795), índice-C 0,777 (0,768–0,783); Columbia (12-derivaçõesd) AUC 0,825 (0,821–0,829), índice-C 0,808 (0,806–0,810) Stanford (DI) AUC 0,797 (0,773–0,824), índice-C 0,781 (0,760–0,793); Cedars-Sinai (DI) AUC 0,778 (0,765–0,792), índice-C 0,771 (0,764–0,778); Columbia (DI) AUC 0,761 (0,757–0,765), índice-C 0,755 (0,753–0,757) Taxa de evento: NR	Stanford (12-derivações) AUC 0,668 (0,647–0,687), índice-C 0,661 (0,652–0,669); Cedars-Sinai (12-derivações) AUC 0,632 (0,591–0,674), índice-C 0,635 (0,619–0,652)Taxa de evento: NR	Stanford HR 3,2 (2,9–3,5) Taxa de evento: NR	-	Stanford HR 2.0 (1.7–2.5) Taxa de evento: NR	Stanford HR 1.6 (1.4–1.9) Taxa de evento: NR	-
Al-Alusi et al.^8^	HR 1,47 (1,36–1,60), Índice-C 0,804 (0,796–0,813) Taxa de evento: 32,7% mortes no grupo de alto risco 13% no grupo de baixo risco	-	-	HR 2,26 (1,90–2,69), Índice-C 0,804 (0,796–0,813) Taxa de evento: 5,9%	-	HR 1,87 (1,69–2,07), Índice-C 0,733 (0,725–0,742) Taxa de evento: 14,1%	HR 1,30 (1,18–1,44) Taxa de evento: 6,5%	-
Sau et al.^9^	Homens HR 1,22 (1,19–1,25), mulheres HR 1,17 (1,19–1,31) Taxa de evento: 18,4%	Homens HR 1,00 (0,63–1,58), mulheres HR 1,78 (1,18–2,70) Taxa de evento: 3,7%	-	-	-	-	-	-
Gao et al.^16^	-	-	-	-	ACC: 75% (Coorte A), 71,8% (Coorte B) Taxa de evento: NR	-	-	-
Raghunath et al.^10^	ECG AUC 0,855 Eventos: 14207	-	-	-	-	-	-	-
Dhingra et al.^11^	YNHHS HR 1,19 (1,15–1,24) UKB HR 2.13 (1,41–3,24) ELSA HR 3,64 (2,27–5,83) Taxa de evento: YNHHS 7,5%, UKB 0,8%, ELSA 1,7%	-	-	YNHHS HR 6,51 (6,11–6,93), índice-C 0,718 (0,697–0,738); UKB HR 18,33 (9,90–33,97), índice-C 0,769 (0,670–0,867); ELSA HR 32,06 (15,36–66,92), índice-C 0,810 (0,714–0,907) Taxa de evento: YNHHS 4,2%	-	YNHHS HR 1,44 (1,04–2,00) UKB HR 3,16 (1,98–5,02) ELSA HR 3,53 (1,4–8,85) Taxa de evento: YNHHS 0,1%, UKB 0,5%, ELSA 0,4%	UKB HR 2,30 (1,36–3,9) ELSA HR 5,74 (2,59–12,72) Taxa de evento: YNHHS 1,6%, UKB 0,5%, ELSA 0,4%	YNHHS HR 2,10 (2,04–2,17) UKB HR 2,79 (2,17–3,6) ELSA HR 4,04 (2,77–5,89) Taxa de evento: YNHHS 10,4%, UKB 1,8%, ELSA 2,5%
Lin et al.^12^	AUROC 0,89, Sensibilidade 0,81, Especificidade 0,83, AUROC (Externa) 0,83 Taxa de evento: 6,3% (3 meses), 8,5% (6 meses), 10,0% (9 meses), 11,4% (1 ano)	-	-	AUROC 0,90 Sensibilidade 0,83 Especificidade 0,81 Taxa de evento: 0,6% (3 meses), 0,8% (6 meses), 1,1% (9 meses), 1,2% (1 ano)	-	AUROC 0,85 Sensibilidade 0,76 Especificidade 0,79 Taxa de evento: 0,3% (3 meses), 0,4% (6 meses), 0,5% (9 meses), 0,5% (1 ano)	AUROC 0,76 Sensibilidade 0,75 Especificidade 0,63 Taxa de evento: 0,3% (3 meses), 0,4% (6 meses), 0,6% (9 meses), 0,7% (1 ano)	-
Butler et al.^13^	-	-	-	ARIC AUC 0,76 (0,72–0,83) MESA AUC 0,77 (0,74–0,79) Taxa de evento: 3,6% (MESA), 5,5% (ARIC)	-	-	-	-
Sun et al.^14^	30 dias: AUROC 0,843 (0,832–0,858), Brier Score 5,97 (5,83–6,12); 1 ano: AUROC 0,812 (0,807–0,81,6), Brier Score 11,44 (11,3-11,6); 5 anos: AUROC 0,798 (0,791–0,802), Brier Score 17,52 (17,29–17,83) Taxa de evento: 7,6% (30 dias) 17,3%, (1 ano) 32,9%, (5 anos)	-	-	-	-	-	-	-
Lin et al.^15^	CGMH: Índice C 0,860 (0,859–0,861) TSGH: Índice C 0,813 (0,807–0,814) Taxa de evento: CGMH 5,9% (3 meses), 7,8% (6 meses), 9,3% (9 meses), 10,5% (1 ano); TSGH 6,8% (3 meses), 8,5% (6 meses), 9,5% (9 meses), 10,5% (1 ano)	CGMH: índice-C 0,891 (0,890–0,893) Taxa de evento: NR	-	-	-	-	-	-

Os valores são apresentados como estimativas pontuais com intervalos de confiança de 95% entre parênteses, salvo indicação em contrário. ACC: acurácia; ARIC: Atherosclerosis Risk in Communities Study (Estudo de Risco de Aterosclerose em Comunidades); DCVA: doença cardiovascular aterosclerótica; AUC/AUROC: área sob a curva ROC; CGMH: Chang Gung Memorial Hospital; ECG: eletrocardiograma; ELSA-Brasil: Estudo Longitudinal de Saúde do Adulto; ICFEp: insuficiência cardíaca com fração de ejeção preservada; HR: hazard ratio (razão de risco); IAM: infarto agudo do miocárdio; MACE: eventos cardiovasculares adversos maiores; MESA: Multi-Ethnic Study of Atherosclerosis (Estudo Multiétnico de Aterosclerose); NR: não reportado; SEER: Surveillance, Epidemiology, and End Results (base de dados dos EUA); TSGH: Tri-Service General Hospital; UKB: UK Biobank (Biobanco do Reino Unido); YNHHS: Yale New Haven Health System

### Mortalidade por todas as causas

Oito estudos relataram resultados promissores na predição de mortalidade por todas as causas utilizando diversos modelos de IA aplicados ao ECG. Lin et al.^12^ demonstraram alta acurácia, com AUROC de 0,89 para mortalidade em 1 ano e 0,83 para mortalidade por todas as causas em 1 ano na validação externa. Sun et al.^14^ encontraram AUROCs de 0,843 para mortalidade em 30 dias, 0,812 para mortalidade em 1 ano e 0,798 para mortalidade em 5 anos, indicando desempenho robusto em múltiplos horizontes temporais. Raghunath et al.^10^ relataram uma AUC de 0,855 utilizando apenas dados de ECG e 0,876 quando idade e sexo foram incorporados. No estudo Surv-ECG, Lin et al.^15^ alcançaram um índice-C de 0,860 para mortalidade por todas as causas. Sau et al.^6^ relataram um índice-C de 0,775, reforçando ainda mais o potencial preditivo dos modelos baseados em IA para risco de mortalidade.

Sau et al.^9^ identificaram HR de 1,22 para homens e 1,17 para mulheres, sugerindo que a discordância entre sexo biológico e sexo estimado pelo ECG pode atuar como um marcador inovador de risco cardiovascular não reconhecido. Corroborando esses achados, Dhingra et al.^11^ relataram um HR de 1,19 para mortalidade por todas as causas utilizando um modelo de IA aplicado a imagens de ECG, em um estudo multinacional que também demonstrou valor preditivo para outros desfechos cardiovasculares, incluindo IAM e IC. Al-Alusi et al.^8^ mostraram que indivíduos classificados como alto risco pelo modelo HTN-AI apresentaram uma taxa de mortalidade em 10 anos significativamente maior (21,0%) em comparação ao grupo de baixo risco (5,4%), destacando o valor da IA na identificação de indivíduos de maior risco.

Houve marcada heterogeneidade entre os estudos, com populações variando de 286 880 a quase 5 milhões de ECGs e um tempo médio de seguimento entre 3 e 34 anos. Embora vários estudos tenham relatado validação externa, na maioria dos casos tratava-se de replicações dentro do mesmo sistema de saúde ou biobanco. Apenas uma minoria testou seus modelos em populações verdadeiramente independentes, com características demográficas e clínicas distintas.^6,11^

### Morte cardiovascular

A capacidade dos modelos de IA aplicados ao ECG para prever mortalidade cardiovascular também foi validada. Sau et al.^6^ relataram um índice-C de 0,832 para a predição de morte cardiovascular. Em Hughes et al.,^7^ o modelo apresentou uma AUC de 0,83 e um índice-C de 0,82 para morte cardiovascular em 5 anos utilizando o ECG de 12 derivações completo, com AUC de 0,80 quando apenas a Derivação I foi utilizada. Sau et al.^9^ relataram um HR de 1,78 para mulheres em comparação a 1,00 para homens entre indivíduos com ECG normal, reforçando o papel da discordância de sexo como um potencial preditor independente de mortalidade cardiovascular, particularmente em mulheres. Lin et al.,^15^ no estudo Surv-ECG, reforçaram ainda mais essa evidência ao relatar um índice-C de 0,891 para predição de morte cardiovascular.

Os estudos que relataram morte cardiovascular também apresentaram heterogeneidade significativa. As populações diferiram em tamanho e duração do seguimento. As taxas de eventos não foram especificadas na maioria dos estudos, e muitas validações classificadas como externas foram conduzidas em coortes derivadas do mesmo sistema de saúde ou de sistemas intimamente relacionados, levantando preocupações quanto à independência dessas validações.

### Doença cardiovascular aterosclerótica

Os modelos de IA aplicados ao ECG também se mostraram eficazes na predição de DCVA. Sau et al.^6^ relataram um índice-C de 0,696 utilizando ECGs de 12 derivações e derivação I, integrados a dados clínicos e genéticos do UK Biobank. Em Hughes et al.,^7^ o modelo alcançou uma AUC de 0,67 para ECG de 12 derivações e 0,63 para Derivação I. No entanto, a predição de eventos de DCVA apresentou ampla variação entre os estudos em termos de tamanho amostral e definições de evento (por exemplo, desfechos compostos incluindo IAM, AVC e morte súbita versus definições mais restritas). Quanto à validação, a maioria ocorreu dentro de instituições ou bases de dados relacionadas, e apenas Sau et al.^6^ realizaram validação externa verdadeiramente independente.

### Infarto do miocárdio

Os modelos de IA aplicados ao ECG demonstraram desempenho promissor na predição de risco de infarto agudo do miocárdio (IAM). Hughes et al.^7^ relataram um AUROC de 0,85 para predição de IAM utilizando o escore SEER, além de um HR de 2,0. De forma semelhante, Al-Alusi et al.^8^ demonstraram um HR de 1,87. Dhingra et al.^11^ também relataram risco elevado, com HR de 1,44 para IAM incidente. Lin et al.^12^ igualmente demonstraram alta acurácia, com AUROC de 0,85.

Os estudos apresentaram heterogeneidade em seu delineamento, com diferenças nas definições de evento, taxas de eventos e tamanhos amostrais variando de milhares a milhões de participantes. Em relação à validação, a maioria permaneceu dentro dos mesmos biobancos ou instituições, e apenas Dhingra et al.^11^ testaram seus modelos em coortes geograficamente distintas.

### Insuficiência cardíaca

Os modelos de IA aplicados ao ECG demonstraram desempenho robusto na predição de IC, embora os estudos apresentem variações substanciais em metodologia. O melhor desempenho foi observado em Lin et al.,^12^ que relataram um AUROC de 0,90 para predição de IC. Dhingra et al.^11^ encontraram HRs de 6,51 na coorte YNHHS, 18,33 no UK Biobank e 32,06 na coorte ELSA-Brasil para IC incidente, demonstrando forte capacidade prognóstica em diferentes populações. Em Sau et al.,^6^ foi alcançado um índice-C de 0,787 utilizando ECGs de 12 derivações e Derivação I, destacando a confiabilidade do modelo.

O modelo de IA-ECG desenvolvido por Butler et al.^13^ alcançou uma AUC de 0,76 na coorte ARIC e 0,77 na coorte MESA utilizando apenas dados de ECG, com melhora significativa quando variáveis clínicas foram incorporadas. Quando combinado com 12 fatores de risco, o modelo AI-ECG-Cox atingiu uma AUC de 0,82 no estudo ARIC e 0,84 no MESA, demonstrando maior valor preditivo por meio da integração de dados clínicos. Em Hughes et al.,^7^ o modelo apresentou sensibilidade de 76% e especificidade de 82% para predição de IC, reforçando suas capacidades diagnósticas. Al-Alusi et al.^8^ relataram um HR de 2,26. Por fim, Gao et al.^16^ relataram acurácia de 75% na Coorte A e 71,8% na Coorte B para insuficiência cardíaca com fração de ejeção preservada, com sensibilidade (71,7%) e especificidade (71,9%) equilibradas.

A maioria das validações foi conduzida em bases de dados relacionadas; apenas um pequeno número de estudos^6,11^ validou seus modelos em populações demograficamente distintas.

### Acidente vascular cerebral (AVC)

Lin et al.^12^ demonstraram o potencial dos modelos de IA aplicados ao ECG na predição de risco de AVC, alcançando um AUROC de 0,76. Em Hughes et al.,^7^ indivíduos da coorte de Stanford com os maiores escores SEER apresentaram um aumento de 1,6 vez no HR para AVC incidente, ajustado por idade e sexo, destacando a capacidade do modelo de identificar precocemente indivíduos com risco cerebrovascular elevado. Dhingra et al.^11^ relataram um HR de 2,30 no UK Biobank, e Al-Alusi et al.^8^ relataram um HR semelhante de 2,30.

Os estudos variaram quanto à inclusão apenas de AVC isquêmico ou de todos os subtipos de AVC, o que contribuiu para diferenças nas taxas de eventos. A validação foi frequentemente limitada a replicações internas ou a bases de dados de instituições relacionadas, e apenas Dhingra et al.^11^ realizaram validação externa verdadeiramente independente.

### Correlação com escores tradicionais

Conforme apresentado na Tabela 3, Lin et al.^12^demonstraram discriminação superior em comparação ao Framingham Risk Score (FRS) em múltiplos desfechos. Dhingra et al.^11^ mostraram que seu modelo de IA-ECG superou a *Pooled Cohort Equation* para insuficiência cardíaca (PCE-HF). Na coorte YNHHS, o modelo de IA-ECG alcançou um índice-C de 0,718, em comparação com 0,601 para o PCE-HF. Sau et al.^6^ relataram que seu modelo de IA-ECG superou o PCE-HF na predição de DCVA, com um índice-C de 0,696 versus 0,547 para o modelo SEER.

**Tabela 3:** – Resumo das associações com escores clínicos tradicionais

Autores	Estudo	Correlação com Escores Tradicionais
Sau et al.^6^	Artificial intelligence-enabled electrocardiogram for mortality and cardiovascular risk estimation: a model development and validation study	A IA-ECG (índice-C 0,696) superou o SEER (índice-C 0,547) e apresentou melhor discriminação em comparação com modelos tradicionais baseados em risco.
Hughes et al.^7^	A deep learning-based electrocardiogram risk score for long term cardiovascular death and disease	Correlação com o PCE (Pearson r = 0,218); NRI: 17,8%; reclassificou 16% dos pacientes de baixo para moderado risco de DCVA (definido pelo PCE). Melhor discriminação para mortalidade cardiovascular em comparação ao PCE, porém menor discriminação para desfechos de DCVA
Al-Alusi et al.^8^	A deep learning digital biomarker to detect hypertension and stratify cardiovascular risk from the electrocardiogram	Discriminação comparável ao PCP-HF para predição de insuficiência cardíaca (índice-C 0,804 vs. 0,806). Discriminação superior ao PCE para IAM (0,733 vs 0,681) e AVC (0,704 vs 0,683)
Sau et al.^9^	Artificial intelligence-enhanced electrocardiography for the identification of a sex-related cardiovascular risk continuum: a retrospective cohort study	NR
Gao et al.^16^	Electrocardiograph analysis for risk assessment of hear t failure with preserved ejection fraction: A deep learning model	NR
Raghunath et al.^10^	Prediction of mortality from 12-lead electrocardiogram voltage data using a deep neural network	O modelo IA-ECG (AUC 0,876) superou o FRS (AUC 0,648) e o CCI (AUC 0,816)
Dhingra et al.^11^	Heart failure risk stratification using artificial intelligence applied to electrocardiogram images: a multinational study	YNHHS: índice-C 0,718 (IA-ECG) vs. 0,601 (PCP-HF); melhor discriminação e reclassificação (NRI 21,9%)
Lin et al.^12^	A multitask deep learning model utilizing electrocardiograms for major cardiovascular adverse events prediction	O modelo IA-ECG demonstrou discriminação superior em comparação ao FRS em todos os desfechos (AUROC em 1 ano: IC 0,90 vs 0,64; IAM 0,85 vs 0,69; AVC 0,76 vs 0,61; mortalidade por todas as causas 0,89 vs 0,52)
Butler et al.^13^	A generalizable electrocardiogram-based artificial intelligence model for 10-year heart failure risk prediction	O modelo IA-ECG foi comparável ao FHS-HF (AUC 0,77 vs 0,74), com desempenho aprimorado quando combinado a variáveis clínicas (AUC 0,84)
Sun et al.^14^	Towards artificial intelligence-based learning health system for population-level mortality prediction using electrocardiograms	NR
Lin et al.^15^	ECG-surv: A deep learning-based model to predict time to 1-year mortality from 12-lead electrocardiogram	O IA-ECG superou modelos de Cox baseados em Framingham para morte cardiovascular (índice-C 0,891 vs 0,734)

IA: inteligência artificial; DCVA: doença cardiovascular aterosclerótica; AUC/AUROC: área sob a curva ROC; CCI: Índice de Comorbidades de Charlson; índice-C: índice de concordância; ECG: eletrocardiograma; FRS: Framingham Risk Score (Escore de Risco de Framingham); FRS-HF: Framingham Risk Score para Insuficiência Cardíaca; HR: hazard ratio (razão de risco); IAM: infarto do miocárdio NRI: melhoria líquida de reclassificação; NR: não reportado; PCE: pooled cohort equations (equações de coorte agrupadas); PCE-HF: pooled cohort equations para prevenção de insuficiência cardíaca; SEER: Surveillance, Epidemiology, and End Results (base de dados dos EUA).

Raghunath et al.^10^ demonstraram desempenho superior na predição de mortalidade por todas as causas em 1 ano, superando tanto o FRS (AUC 0,648) quanto o *Charlson Comorbidity Index* (CCI) (AUC 0,816), alcançando uma AUC de 0,876 utilizando apenas dados de ECG. Em Butler et al.,^13^ o FRS para insuficiência cardíaca apresentou AUC de 0,78 na coorte ARIC e 0,74 na coorte MESA. Hughes et al.^7^ encontraram correlação significativa com o PCE-HF, relatando uma *Net Reclassification Improvement* (NRI) de 17,8%, reclassificando corretamente 16% dos pacientes inicialmente categorizados como baixo risco para a categoria de risco moderado.

### Qualidade dos estudos

A qualidade dos estudos incluídos foi avaliada utilizando a escala Newcastle–Ottawa (NOS), conforme apresentado na Tabela 4. A NOS avalia os estudos em três domínios: seleção, comparabilidade e desfecho. Cada domínio contém critérios específicos, com uma pontuação máxima possível de 9 pontos.

**Tabela 4:** – Avaliação da qualidade dos estudos pela Escala de Newcastle-Ottawa

Estudo	Seleção				Comparabilidade		Desfecho			Escore total de qualidade
	Representatividade da Coorte Exposta	Seleção da Coorte Não Exposta	Determinação da Exposição	Desfecho Ausente no Início do Estudo	Fator Principal	Fator adicional	Avaliação dos Desfechos	Duração do Seguimento	Adequação do Seguimento	
Sau et al.^6^	*	*	*	*	*	*	*	*	*	9
Hughes et al.^7^	*	*	*	*	*	*	*	*	*	9
Al-Alusi et al.^8^	*	*	*	*	*	*	*	*	*	9
Sau et al.^9^	*	*	*		*	*	*	*	*	8
Gao et al.^16^	*	*	*		*	*	*	*	*	8
Raghunath et al.^10^	*	*	*	*	*	*	*	*	*	9
Dhingra et al.^11^	*	*	*	*	*	*	*	*	*	9
Lin et al.^12^	*	*	*	*	*	*	*	*	*	9
Butler et al.^13^	*	*	*	*	*	*	*	*	*	9
Sun et al.^14^	*		*	*	*	*	*	*	*	8
Lin et al.^15^	*	*	*	*	*	*	*	*	*	9

Oito estudos alcançaram a pontuação máxima de 9, indicando alta qualidade metodológica. Esses estudos demonstraram forte seleção de coorte, comparabilidade adequada com base em fatores-chave e avaliação robusta dos desfechos, com períodos de seguimento apropriados. Três estudos obtiveram 8 pontos devido à ausência de informações sobre fatores de comparabilidade ou à falta de confirmação de que o desfecho não estava presente no início do estudo. No entanto, vários estudos não relataram características demográficas essenciais, como idade, sexo ou etnia – fatores importantes para avaliar a validade externa e a equidade entre subgrupos. Embora essas omissões não sejam totalmente capturadas pela NOS, elas representam limitações relevantes ao considerar a generalização do desempenho de modelos de IA em populações diversas. Apesar dessas questões, a qualidade geral dos estudos foi alta, sustentando a confiabilidade dos achados da revisão.

### Técnicas de Interpretabilidade em Modelos de IA-ECG

Entre os estudos incluídos, técnicas de interpretabilidade foram relatadas em nove dos onze artigos, conforme mostrado na Tabela 5. Os *saliency maps* foram os métodos mais frequentemente aplicados para destacar as regiões do traçado de ECG mais relevantes para as predições dos modelos (por exemplo, onda P, intervalo PR).^8,10,12,14,15^ Outras estratégias incluíram *SHapley Additive exPlanations* (SHAP),^13,14^ que forneceram atribuição de características em modelos baseados em árvores ou CNN; ablação de neurônios e *heatmaps* de pesos de CNN,^16^ que examinaram o comportamento interno do modelo por meio de perturbações; além de autoencoders variacionais (VAEs), média de formas de onda e análises de associação biológica (GWAS, PheWAS) em Sau et al.^6^ e Sau et al.,^9^utilizadas para explorar plausibilidade biológica e padrões de morfologia do ECG. Dois estudos não relataram o uso de qualquer técnica de interpretabilidade.^7,11^

**Tabela 5:** – Técnicas de interpretabilidade aplicadas nos estudos incluídos

Estudo	Técnicas de Interpretabilidade	Descrição
Sau et al.^6^	VAE, média de formas de onda, PheWAS, GWAS, modelos de Cox	Vinculou as predições da IA-ECG a características biológicas e mecanismos fisiopatológicos plausíveis
Hughes et al.^7^	NR	Comparou o desempenho com os escores de risco PCE e SEER, mas não relatou métodos específicos de interpretabilidade
Al-Alusi et al.^8^	Mapas de saliência	Utilizado para identificar segmentos da onda do ECG preditivos de hipertensão
Sau et al.^9^	VAE, média de formas de onda	Visualizou a morfologia do ECG associada à discordância de risco baseada em sexo; VAE utilizado para redução de dimensionalidade
Gao et al.^16^	Ablação de neurônios, mapas de calor de pesos da CNN	Perturbou neurônios nas camadas da CNN e visualizou os pesos associados; abordagem semelhante à análise de sensibilidade por oclusão
Raghunath et al.^10^	Mapas de saliência, feedback de especialistas	Forneceu explicações visuais para ECGs normais sinalizados pela IA; incluiu feedback de cardiologistas para avaliar interpretabilidade
Dhingra et al.^11^	NR	Utilizou IA baseada em imagens de ECG para predição de risco; não relatou métodos específicos de interpretabilidade
Lin et al.^12^	Mapas de saliência	Identificou a onda P e o intervalo PR como principais preditores de mortalidade usando visualização de saliência
Butler et al.^13^	SHAP	Aplicou SHAP a características derivadas do ECG para estratificação de risco de insuficiência cardíaca; identificou os traços de ECG mais relevantes
Sun et al.^14^	SHAP, mapas de saliência	SHAP utilizado no XGB para atribuição de características; Grad-CAM aplicado à CNN para destacar segmentos-chave do ECG (PR, QRS, ST-T)
Lin et al.^15^	mapas de saliência	Aplicou mapas de saliência visual para interpretação do modelo

CNN: rede neural convolucional; ECG: eletrocardiograma; Grad-CAM: Gradient-weighted Class Activation Mapping (mapeamento de ativação de classe ponderado por gradiente); GWAS: Genome-Wide Association Study (estudo de associação genômica ampla); IA: inteligência artificial; NR: não reportado; PCE: Pooled Cohort Equation (equação de coorte agrupada); PheWAS: Phenome-Wide Association Study (estudo de associação fenotípica ampla); PR: intervalo PR; QRS: complexo QRS; SEER: Surveillance, Epidemiology, and End Results (base de dados dos EUA); SHAP: SHapley Additive exPlanations; ST-T: segmento ST e onda T; VAE: Variational Autoencoder (autoencoder variacional); XGB: XGBoost.

### Aplicação clínica e uso prospectivo da IA-ECG

Quanto à aplicação clínica, a maioria dos estudos explorou o potencial dos modelos de IA-ECG para estratificação precoce de risco, monitoramento de pacientes ou triagem em nível populacional; entretanto, essas aplicações permaneceram retrospectivas ou teóricas. Notavelmente, apenas Gao et al.^16^ conduziram uma aplicação prospectiva de seu modelo de DL: o algoritmo foi aplicado no momento da admissão de novos pacientes com suspeita de insuficiência cardíaca com fração de ejeção preservada, e as predições foram comparadas com medidas invasivas de pressão diastólica final do ventrículo esquerdo. Esse cenário de validação em tempo real representa o único exemplo de uso do modelo em um contexto de tomada de decisão clínica entre os estudos incluídos. Embora constitua o único cenário de implementação em tempo real, ele não con validação prospectiva para predição de desfechos clínicos.

## Discussão

Os estudos revisados demonstram avanços substanciais na aplicação de IA à análise de ECG para estratificação de risco cardiovascular. Técnicas de DL, particularmente CNNs, mostraram capacidade de detectar alterações sutis no ECG associadas à hipertensão, disfunção ventricular e fibrilação atrial — mesmo em traçados considerados normais por especialistas. Esses achados reforçam o potencial clínico da IA-ECG para a detecção precoce de condições assintomáticas ou subclínicas, com a possibilidade de transformar a prática clínica ao permitir, a partir de um único exame, a identificação de indivíduos de alto risco e a implementação de intervenções precoces.

Paralelamente, outras linhas importantes de investigação têm explorado biomarcadores derivados de ECG por IA, como a idade eletrocardiográfica, que demonstraram fortes associações com mortalidade e desfechos cardiovasculares em grandes coortes populacionais. Por exemplo, estudos anteriores mostraram que discrepâncias entre a idade estimada pelo ECG por IA e a idade cronológica estão associadas a maior risco de mortalidade e eventos cardiovasculares incidentes, incluindo achados provenientes de coortes comunitárias.^18-20^ Essas abordagens destacam o potencial mais amplo da IA-ECG para capturar sinais latentes de envelhecimento fisiológico e biológico. Embora tais estudos não tenham sido incluídos na presente revisão devido ao nosso foco pré-definido em modelos que predizem diretamente desfechos clínicos a partir exclusivamente dos sinais de ECG, eles fornecem evidências complementares importantes que sustentam o valor prognóstico da IA-ECG.

Apesar desses avanços, importantes limitações metodológicas comprometem comparações diretas entre os estudos. A heterogeneidade dos modelos de IA utilizados, a diversidade de métricas de desempenho empregadas e a variação nos desfechos clínicos analisados dificultam a consolidação dos resultados. Uma consideração crucial diz respeito à distinção entre validade estatística e validade clínica: embora métricas como AUROC e HR indiquem alto desempenho, permanece essencial determinar se as anormalidades identificadas pelos modelos correspondem a fenótipos fisiologicamente significativos, com implicações práticas para o manejo clínico. Caso contrário, existe o risco de que os algoritmos detectem apenas assinaturas estatísticas sem relevância clínica real.

A análise dos resultados mostra que, para mortalidade por todas as causas, os estudos utilizaram diferentes métricas – como AUROC e HR – além de períodos de seguimento variados (por exemplo, mortalidade em 1 ano versus 5 anos), o que dificulta comparações diretas. Ainda assim, nos estudos que utilizaram as mesmas métricas, os modelos demonstraram desempenho consistente em todos os horizontes temporais. Limitação semelhante foi observada para mortalidade cardiovascular, embora todos os modelos tenham apresentado alta acurácia preditiva. Apenas dois estudos avaliaram DCVA, cada um empregando métricas distintas, o que limitou comparações diretas; apesar de discriminação ligeiramente menor em comparação com mortalidade por todas as causas, os resultados permaneceram satisfatórios. Para infarto agudo do miocárdio, quatro estudos relataram desfechos: três utilizando valores de HR variando de 1,44 a 3,53 em diferentes coortes, e dois relatando AUROC em torno de 0,85, indicando desempenho preditivo consistente. Insuficiência cardíaca esteve entre os desfechos com maior desempenho relatado, alcançando AUROC de 0,90 e HR de 32,06. Por fim, alguns estudos também avaliaram acidente vascular cerebral, relatando HR entre 1,6 e 2,3, reforçando a capacidade dos modelos de IA-ECG de capturar risco em múltiplos desfechos cardiovasculares.

Modelos de IA-ECG tendem a apresentar desempenho mais robusto para mortalidade por todas as causas em comparação com desfechos cardiovasculares mais específicos, como DCVA. Essa diferença provavelmente reflete o fato de que a mortalidade por todas as causas abrange um espectro mais amplo de anormalidades sistêmicas e cardíacas que podem ser detectáveis nos sinais de ECG, enquanto a doença aterosclerótica nem sempre produz alterações elétricas ou estruturais mensuráveis antes da ocorrência de eventos clínicos. Como resultado, a aterosclerose subclínica pode permanecer indetectável por modelos baseados em ECG, limitando seu desempenho preditivo para DCVA. Além disso, a predição de DCVA apresentou heterogeneidade substancial entre os estudos, incluindo diferenças no tamanho das amostras e nas definições de desfecho, o que restringe ainda mais a comparabilidade direta e pode contribuir para a variabilidade dos resultados relatados.

Comparações entre modelos de IA-ECG e escores tradicionais de risco devem ser interpretadas com cautela, pois muitas ferramentas convencionais não foram originalmente desenvolvidas para prever desfechos como mortalidade por todas as causas. Diferenças nos alvos de predição podem explicar parcialmente as variações nas métricas de desempenho relatadas e limitar comparações diretas. Do ponto de vista clínico, esses achados sugerem que modelos de IA-ECG e escores tradicionais de risco podem desempenhar papéis complementares: enquanto ferramentas convencionais permanecem mais adequadas para estimar risco específico de doença (por exemplo, DCVA), a IA-ECG pode oferecer uma estratificação de risco mais ampla ao capturar sinais fisiológicos globais, apoiando uma avaliação mais integrada do risco do paciente.

Apesar disso, persistem lacunas metodológicas. Nem todos os estudos relataram o número de eventos clínicos, alguns omitiram métricas de calibração e muitos concentraram-se em desfechos de curto prazo, o que limita a extrapolação para risco de longo prazo. Outro aspecto pouco explorado é a generalização temporal: a maioria dos modelos foi desenvolvida em coortes estáticas, sem avaliar se o desempenho permanece estável ao longo do tempo em populações sujeitas a mudanças epidemiológicas, terapêuticas ou de estilo de vida. A atualização contínua dos modelos e o monitoramento prospectivo serão necessários para garantir utilidade clínica duradoura da IA-ECG.

Além disso, nossa seleção de estudos foi restrita a modelos que utilizam sinais de ECG como única variável de entrada, o que aumenta a consistência, mas não reflete completamente a prática clínica real, na qual múltiplos fatores do paciente são considerados. Portanto, quando combinada com variáveis clínicas tradicionais, a IA-ECG tem potencial para funcionar como um biomarcador digital, aprimorando a identificação de indivíduos de alto risco e reduzindo o subdiagnóstico. Pesquisas futuras devem avançar integrando IA-ECG com dados laboratoriais, exames de imagem e até informações provenientes de dispositivos vestíveis, caminhando em direção a uma abordagem multimodal capaz de personalizar a estratificação de risco cardiovascular.

A escalabilidade da IA-ECG representa uma grande vantagem, especialmente em contextos com recursos limitados, dada a acessibilidade e a simplicidade da aquisição do ECG. No entanto, a maioria dos modelos foi desenvolvida e validada utilizando dados de países de alta renda, predominantemente dos Estados Unidos, o que limita sua generalização. Em muitos casos, as validações consistiram em replicações dentro de sistemas de saúde relacionados, biobancos ou populações com perfis demográficos e clínicos semelhantes, em vez de avaliações em coortes distintas e heterogêneas. Distinguir entre replicação interna e validação externa genuína é essencial, pois esta última fornece o teste mais rigoroso da robustez e da transportabilidade dos modelos de IA-ECG.

O estudo de Sau et al.^6^ foi um dos poucos a realizar validação externa em uma população geográfica ou demograficamente distinta – especificamente no Brasil – fornecendo evidências mais fortes de generalização. Um segundo estudo, de Dhingra et al.,^11^ também incluiu uma coorte brasileira, confirmando que, mesmo em populações com características sociodemográficas e clínicas diferentes, os modelos mantiveram bom desempenho. Al-Alusi et al.^8^ conduziram validações interna e externa em populações semelhantes, com pequenas diferenças, como maior proporção de mulheres e indivíduos negros na coorte externa. Ainda assim, os perfis de comorbidades eram amplamente comparáveis entre os grupos. Por outro lado, Gao et al.^16^ não relataram características detalhadas da população, limitando as comparações entre coortes. Da mesma forma, nos estudos de Lin et al.^12^ e Lin et al.,^15^ a validação externa foi limitada devido à ausência de informações clínicas, permitindo apenas a avaliação da mortalidade em um ano como desfecho.

O número reduzido de validações verdadeiramente independentes, combinado com dados demográficos incompletos, permanece uma barreira importante para a ampla adoção clínica dessas ferramentas, pois dificulta a avaliação da diversidade populacional e reduz tanto a comparabilidade quanto a interpretabilidade dos resultados.

Entre os pontos fortes dos estudos incluídos estão o uso de grandes bases de dados, metodologias avançadas de IA – especialmente CNNs – e períodos de seguimento prolongados. As CNNs demonstraram notável eficácia na análise de sinais brutos de ECG, capturando características temporais e espaciais com mínima necessidade de pré-processamento. Em contraste, estudos classificados como DNNs ou DL geral frequentemente careciam de especificidade arquitetural e ofereciam interpretabilidade limitada.

Nesse contexto, a maioria dos estudos analisados empregou técnicas de interpretabilidade para aumentar a transparência dos modelos de IA aplicados ao ECG, refletindo a crescente demanda por sistemas explicáveis. O método mais utilizado foi o de *saliency maps*, que gera *heatmaps* sobre o traçado do ECG para destacar regiões presumivelmente relevantes para as predições, como a onda P, o complexo QRS ou o intervalo PR. Apesar de sua popularidade, evidências da literatura em imagem médica indicam que esses mapas podem ser instáveis, pouco reprodutíveis e, em alguns casos, fornecer explicações enganosas que parecem plausíveis, mas não refletem o verdadeiro raciocínio do modelo.^21,22^

O SHAP também foi utilizado em vários estudos. Esse método, fundamentado na teoria dos jogos, atribui valores quantitativos à contribuição de cada variável para a saída do modelo, permitindo quantificar a influência de cada característica. Diferentemente dos métodos puramente baseados em gradiente, o SHAP fornece uma estrutura de atribuição aditiva que integra interpretações locais e globais, oferecendo maior consistência entre ambas. Sua capacidade de gerar visualizações intuitivas — como gráficos coloridos que destacam pixels importantes em imagens ou segmentos temporais relevantes em sinais de ECG — tornou-o particularmente valioso em aplicações biomédicas. Entre suas limitações estão o alto custo computacional, desafios relacionados a variáveis temporais altamente correlacionadas e possíveis inconsistências na captura de dependências entre características.^23,24^

Outras abordagens de interpretabilidade, incluindo ablação de neurônios combinada com *heatmaps* de pesos de CNN, VAEs e análises de associação biológica (por exemplo, GWAS, PheWAS), também foram exploradas para fornecer insights adicionais sobre o comportamento dos modelos. Essas técnicas podem esclarecer representações latentes ou a plausibilidade biológica das predições, mas muitas vezes são limitadas por complexidade técnica, restrições de acesso ao modelo e vieses presentes nos dados.^22-24^

De modo geral, o cenário atual indica que, embora múltiplas técnicas de interpretabilidade estejam disponíveis, sua integração na prática clínica ainda é limitada. Como observado por Yanagawa e Sato,^22^ esforços futuros devem priorizar métodos que não apenas esclareçam as predições dos modelos, mas também aproximem a compreensão clínica dos padrões do ECG, potencialmente revelando marcadores fisiológicos sutis ou subtipos de doença que não são facilmente identificáveis por meio da análise convencional. A combinação de interpretabilidade com validação sistemática e avaliação clínica prospectiva pode transformar os modelos de IA de ferramentas puramente preditivas em instrumentos que apoiam ativamente o raciocínio clínico e a geração de hipóteses.

Notavelmente, apenas Gao et al.^16^ testaram prospectivamente a IA-ECG em um cenário do mundo real, aplicando o modelo no momento da admissão para estimar o risco de ICFEp em comparação com medições invasivas. Embora modelos como o AIRE^6^ e o ECG-surv15^15^ tenham demonstrado fortes associações com características fisiológicas e sobrevida, a implementação clínica em larga escala ainda é incipiente.

Para que a IA-ECG faça a transição do uso experimental para a prática clínica, é essencial sua integração com os sistemas de saúde e os prontuários eletrônicos. Os marcos regulatórios precisam evoluir para avaliar os modelos não apenas quanto à acurácia, mas também quanto à equidade, transparência e utilidade clínica. Investimentos em infraestrutura, interoperabilidade e capacitação de profissionais de saúde são necessários para garantir o uso seguro e eficaz dessas tecnologias. Ensaios clínicos prospectivos também são fundamentais, especialmente em contextos de prevenção primária.

Por fim, estratégias de saúde pública podem se beneficiar da IA-ECG ao possibilitar a detecção precoce de indivíduos de alto risco, potencialmente reduzindo custos de longo prazo e melhorando desfechos. No entanto, considerações éticas – como transparência algorítmica, privacidade de dados, responsabilidade e acesso equitativo – precisam ser abordadas. Modelos treinados em populações homogêneas e privilegiadas correm o risco de perpetuar disparidades em saúde, a menos que sejam adaptados a contextos clínicos e demográficos diversos.

Em resumo, o uso efetivo da IA-ECG exige uma abordagem transdisciplinar que conecte inovação tecnológica, prática clínica, saúde pública e ética. Somente sob essa perspectiva a IA-ECG pode evoluir de uma ferramenta promissora para um instrumento transformador na redução de iniquidades em saúde, na otimização do cuidado e no avanço da saúde populacional.

## Conclusão

A aplicação de IA à análise de ECG representa um avanço promissor na avaliação personalizada do risco cardiovascular. No entanto, mais pesquisas são necessárias para garantir a segurança, a eficácia e a integração clínica equitativa dessas tecnologias. As evidências atuais, embora convincentes, destacam a necessidade de populações mais diversas nos conjuntos de treinamento e validação, maior interpretabilidade dos modelos e ensaios clínicos prospectivos para avaliar plenamente o impacto no mundo real e a generalização dos modelos de IA-ECG. Somente por meio de validação rigorosa e integração cuidadosa a IA-ECG poderá fazer a transição de uma fronteira de pesquisa para uma ferramenta transformadora na medicina cardiovascular.

## References

[1] World Health Organization (2025). Noncommunicable diseases.

[2] Lüscher TF, Wenzl FA, D'Ascenzo F, Friedman PA, Antoniades C (2024). Artificial Intelligence in Cardiovascular Medicine: Clinical Applications. Eur Heart J.

[3] Arnett DK, Blumenthal RS, Albert MA, Buroker AB, Goldberger ZD, Hahn EJ (2019). 2019 ACC/AHA Guideline on the Primary Prevention of Cardiovascular Disease: A Report of the American College of Cardiology/American Heart Association Task Force on Clinical Practice Guidelines. Circulation.

[4] Talha I, Elkhoudri N, Hilali A (2024). Major Limitations of Cardiovascular Risk Scores. Cardiovasc Ther.

[5] Page MJ, McKenzie JE, Bossuyt PM, Boutron I, Hoffmann TC, Mulrow CD (2021). The PRISMA 2020 Statement: An Updated Guideline for Reporting Systematic Reviews. BMJ.

[6] Sau A, Pastika L, Sieliwonczyk E, Patlatzoglou K, Ribeiro AH, McGurk KA (2024). Artificial Intelligence-Enabled Electrocardiogram for Mortality and Cardiovascular Risk Estimation: A Model Development and Validation Study. Lancet Digit Health.

[7] Hughes JW, Tooley J, Soto JT, Ostropolets A, Poterucha T, Christensen MK (2023). A Deep Learning-Based Electrocardiogram Risk Score for Long Term Cardiovascular Death and Disease. NPJ Digit Med.

[8] Al-Alusi MA, Friedman SF, Kany S, Rämö JT, Pipilas D, Singh P (2025). A Deep Learning Digital Biomarker to Detect Hypertension and Stratify Cardiovascular Risk from the Electrocardiogram. NPJ Digit Med.

[9] Sau A, Sieliwonczyk E, Patlatzoglou K, Pastika L, McGurk KA, Ribeiro AH (2025). Artificial Intelligence-Enhanced Electrocardiography for the Identification of a Sex-Related Cardiovascular Risk Continuum: A Retrospective Cohort Study. Lancet Digit Health.

[10] Raghunath S, Cerna AEU, Jing L, vanMaanen DP, Stough J, Hartzel DN (2020). Prediction of Mortality from 12-Lead Electrocardiogram Voltage Data Using a Deep Neural Network. Nat Med.

[11] Dhingra LS, Aminorroaya A, Sangha V, Pedroso AF, Asselbergs FW, Brant LCC (2025). Heart Failure Risk Stratification Using Artificial Intelligence Applied to Electrocardiogram Images: A Multinational Study. Eur Heart J.

[12] Lin CH, Liu ZY, Chu PH, Chen JS, Wu HH, Wen MS (2025). A Multitask Deep Learning Model Utilizing Electrocardiograms for Major Cardiovascular Adverse Events Prediction. NPJ Digit Med.

[13] Butler L, Karabayir I, Kitzman DW, Alonso A, Tison GH, Chen LY (2023). A Generalizable Electrocardiogram-Based Artificial Intelligence Model for 10-Year Heart Failure Risk Prediction. Cardiovasc Digit Health J.

[14] Sun W, Kalmady SV, Sepehrvand N, Salimi A, Nademi Y, Bainey K (2023). Towards Artificial Intelligence-Based Learning Health System for Population-Level Mortality Prediction Using Electrocardiograms. NPJ Digit Med.

[15] Lin CH, Liu ZY, Chen JS, Fann YC, Wen MS, Kuo CF (2025). ECG-Surv: A Deep Learning-Based Model to Predict Time to 1-Year Mortality from 12-Lead Electrocardiogram. Biomed J.

[16] Gao Z, Yang Y, Yang Z, Zhang X, Liu C (2025). Electrocardiograph Analysis for Risk Assessment of Heart Failure with Preserved Ejection Fraction: A Deep Learning Model. ESC Heart Fail.

[17] Lima EM, Ribeiro AH, Paixão GMM, Ribeiro MH, Pinto-Filho MM, Gomes PR (2021). Deep Neural Network-Estimated Electrocardiographic Age as a Mortality Predictor. Nat Commun.

[18] Brant LCC, Ribeiro AH, Pinto-Filho MM, Kornej J, Preis SR, Fetterman JL (2023). Association between Electrocardiographic Age and Cardiovascular Events in Community Settings: The Framingham Heart Study. Circ Cardiovasc Qual Outcomes.

[19] Baek YS, Lee DH, Jo Y, Lee SC, Choi W, Kim DH (2023). Artificial Intelligence-Estimated Biological Heart Age Using a 12-Lead Electrocardiogram Predicts Mortality and Cardiovascular Outcomes. Front Cardiovasc Med.

[20] Bozzi ICRS, Lima MCAG, Ribeiro ALP, Paixão GMM (2026). Artificial Intelligence-Derived ECG-Age as a Predictor of Mortality and Cardiovascular Events: A Systematic Review and Meta-Analysis. Arq Bras Cardiol.

[21] Zhang J, Chao H, Dasegowda G, Wang G, Kalra MK, Yan P (2024). Revisiting the Trustworthiness of Saliency Methods in Radiology AI. Radiol Artif Intell.

[22] Yanagawa M, Sato J (2024). Seeing is Not Always Believing: Discrepancies in Saliency Maps. Radiol Artif Intell.

[23] Band SS, Yarahmadi A, Hsu CC, Biyari M, Sookhak M, Ameri R (2023). Application of Explainable Artificial Intelligence in Medical Health: A Systematic Review of Interpretability Methods. Inform Med Unlocked.

[24] Sun Q, Akman A, Schuller BW Explainable Artificial Intelligence for Medical Applications: A Review.

