# Supplementary material for: Inteligência Artificial Aplicada à Avaliação de Risco Cardiovascular Baseada em Eletrocardiograma: Uma Revisão Sistemática
Source: Arq Bras Cardiol. 2026 Jul 22;123(7):e20250790. [Article in Portuguese] doi: 10.36660/abc.20250790 (PMC13399140; doi:10.36660/abc.20250790)
Supplement: Translation

# Translation: Artificial Intelligence Applied to Electrocardiogram-Based Cardiovascular Risk Assessment: A Systematic Review

**DOI:** 10.36660/abc.20250790i

**Authors:** Maria Clara Mantoan Pinheiro, Lívia Felberg, Isadora Cristine Reis Sguizzato Bozzi, Antônio Luiz Pinho Ribeiro, Gabriela Miana de Mattos Paixão

**Affiliations:** 1 Brazil Universidade Federal de Minas Gerais, Belo Horizonte, MG – Brazil

**Keywords:** Artificial Intelligence, Heart Disease Risk Factors, Electrocardiography

## Abstract

Cardiovascular diseases (CVDs) are the leading cause of mortality worldwide, underscoring the need for effective risk prediction and early detection. Although the electrocardiogram (ECG) is a widely available and low-cost diagnostic tool, its traditional interpretation is limited by subjectivity. Artificial intelligence (AI) has emerged as a promising approach, capable of extracting hidden prognostic information from ECG signals. This systematic review aimed to assess original studies applying AI techniques to ECGs for cardiovascular risk prediction and mortality. Original studies that used ECG signals as the sole input variable for AI models, focusing on cardiovascular risk outcomes, were included. A systematic search was conducted in different databases, and data were synthesized narratively. Eleven studies were included, predominantly retrospective cohorts applying convolutional neural networks (CNNs) to predict cardiovascular risk or mortality. The sample primarily consisted of adult populations in high-income countries. Primary outcomes included all-cause mortality, cardiovascular death, and major adverse cardiovascular events (MACE). Reported AUROC values ranged from 0.63 to 0.961 in training sets, with some models outperforming traditional risk scores. AI-ECG models demonstrated the potential to detect subclinical disease, enabling early risk stratification even in normal ECGs. However, challenges remain regarding population diversity, model interpretability, and prospective validation. The application of AI to ECG analysis represents a promising advancement in personalized cardiovascular risk assessment. Nonetheless, further research is needed to ensure the safety, effectiveness, and equitable clinical integration of these technologies.

## Introduction

Cardiovascular diseases (CVDs) are the leading cause of mortality worldwide, encompassing both communicable and noncommunicable conditions.^1^ Therefore, cardiovascular risk stratification and the early detection of these diseases are essential. In this context, the electrocardiogram (ECG) stands out as a well-established tool due to its high availability, low cost, and operational simplicity. However, ECG interpretation has an important limitation: its subjectivity, as it relies on visual criteria that depend on the examiner.

In recent years, artificial intelligence (AI) has gained significant prominence, particularly in the field of medicine, opening new frontiers in data analysis. Machine learning (ML) and deep learning (DL) approaches have demonstrated the ability to recognize complex and subtle patterns that may go unnoticed by human observers. This has led to the belief that integrating AI with ECG analysis may enable the extraction of prognostic information and cardiovascular risk stratification insights that were previously unidentified.^2,3^

Current models for estimating cardiovascular risk have notable limitations, as they do not always accurately reflect an individual patient’s reality and often do not incorporate ECG data. The use of additional methods, such as coronary calcium scoring, has shown good performance for cardiovascular risk re-stratification; however, cost remains a significant barrier, particularly in low- and middle-income countries.^4^ As a result, a considerable number of patients are underserved, especially those from diverse populations whose social and racial characteristics are not adequately considered. This underscores the need for validation in heterogeneous populations — a practice still lacking in some existing risk stratification tools.^4^

Although most atherosclerotic events can be prevented through health-promotion strategies and the control of known risk factors, individual variables persist, including low treatment adherence and social inequality.^2^ In this context, applying AI to ECG analysis may offer an alternative to support the development of more sensitive, personalized, and integrated prediction models.^3^

The purpose of this article is to provide an up-to-date and critical systematic review of the primary applications of AI in ECG interpretation, with a focus on predicting major adverse cardiovascular events (MACE). It examines recent advances, the challenges associated with large-scale implementation, and the prospects for adoption in clinical practice. The main findings of this systematic review are summarized in the Central Illustration.

## Methods

This systematic review was conducted in accordance with the Preferred Reporting Items for Systematic Reviews and Meta-Analyses (PRISMA) guidelines.^5^ The protocol was registered in PROSPERO (1039916).

The PICOTS framework (Population, Intervention, Comparator, Outcome, Timing, and Study design) guided the methodology of this systematic review. Studies involving adult populations that applied AI models using only ECG signals as input, without requiring an explicit comparator group, were included. The primary outcomes were all-cause mortality, cardiovascular death, heart failure (HF), myocardial infarction (MI), stroke, and atherosclerotic cardiovascular disease (ASCVD). Follow-up periods varied across studies, with a predominance of retrospective cohort designs.

### Eligibility criteria

We included original research articles published in English within the past five years that directly evaluated the use of AI – specifically convolutional neural networks (CNNs) or deep neural networks (DNNs) – applied exclusively to ECGs for predicting cardiovascular risk in adult populations (aged ≥18 years). Eligible studies assessed the risk or prediction of all-cause mortality, ASCVD, and major adverse cardiovascular events (MACE), including MI, HF, stroke, and cardiovascular death.

We excluded studies that: (1) integrated ECG data with other clinical, laboratory, or imaging information; (2) focused exclusively on arrhythmia diagnosis; (3) were limited to specific patient populations; or (4) were narrative reviews, case reports, or did not provide access to the full text.

### Information sources and search strategy

The literature search was conducted in PubMed, Cochrane Library, Scopus, and ScienceDirect. Searches were limited to articles published between 2020 and 2025, and the final search was performed in May 2025. The following English-language terms and their synonyms were used as search descriptors: “Artificial Intelligence,” “Machine Learning,” “Deep Learning,” “Electrocardiography,” “Electrocardiogram,” “ECG,” “Cardiovascular Risk Factors,” “Risk Assessment,” “Cardiovascular Risk,” and “Heart Disease Risk.” Boolean operators “AND” and “OR” were applied to combine terms.

### Selection process

The selection process consisted of four stages: identification, screening, eligibility assessment, and inclusion. The initial search identified 1,118 records, of which 1,056 were excluded after title screening because they did not address the core focus of the review. Of the remaining 62 records, 45 were excluded following abstract assessment and removal of duplicates. Subsequently, six additional records were excluded after full-text evaluation for not meeting the inclusion criteria. Ultimately, eleven studies fulfilled all eligibility criteria and were included in the review.

Two reviewers independently conducted all phases of the selection process. Any discrepancies were resolved through discussion with a third reviewer. The complete selection workflow is presented in the PRISMA flow diagram (Figure 1).

**Figure 1:** – PRISMA Flow Diagram: study selection process.

### Data collection process and data Items

Data extraction was performed independently by two reviewers using standardized data extraction forms created in Microsoft Excel. Extracted data included study design, population characteristics, AI model type (CNN or DNN), ECG acquisition details, and the cardiovascular outcomes assessed. Additional variables collected included study setting, sample size, the training and validation procedures of the AI models, and whether external validation was conducted.

## Results

A total of eleven original studies were included in this systematic review. Their characteristics are summarized in Table 1. Ten studies were retrospective cohorts^6-15^ and one study had a prospective design.^16^ The studies analyzed a wide range of ECG signals, predominantly 12-lead ECGs, with some also evaluating single-lead (Lead I) recordings.^6,7^

**Table 1:** – Characteristics of Included AI-ECG Studies

Authors	Year of publication	Article Title	Study Type	AI Techniques	Signal Input Type	Training Population	Number of ECGs	Mean Age (years)	Male (%)	Follow-up (Mean, years)	External Validation	Validation Population
Sau et al.^6^	2024	Artificial intelligence-enabled electrocardiogram for mortality and cardiovascular risk estimation: a model development and validation study	Retrospective	CNN	12-lead and single-lead (Lead I) ECG	BIDMC (USA)	1,163,401	NR	NR	5.46	Yes	CODE (Brazil) SaMi-Trop (Brazil) ELSA-Brazil (Brazil) UK Biobank (UK)
Hughes et al.^7^	2023	A deep learning-based electrocardiogram risk score for long term cardiovascular death and disease	Retrospective	CNN	12-lead and single-lead (Lead I) ECG	Stanford Health Care (USA)	500,000	59	54%	5	Yes	Cedars-Sinai Medical Center (USA) Columbia University Irving Medical Center (USA)
Al-Alusi et al.^8^	2025	A deep learning digital biomarker to detect hypertension and stratify cardiovascular risk from the electrocardiogram	Retrospective	CNN	12-lead ECG	Massachusetts General Hospital (USA)	752,415	57.3 ± 16.8	51.6%	5.6–7.8	Yes	Brigham and Women’s Hospital (USA)
Sau et al.^9^	2025	Artificial intelligence-enhanced electrocardiography for the identification of a sex-related cardiovascular risk continuum: a retrospective cohort study	Retrospective	CNN	12-lead ECG	BIDMC (USA)	1,163,401	57.7 ± 18.69	47.9%	3.4–4.8	Yes	UK Biobank (UK)
Gao et al.^16^	2025	Electrocardiograph analysis for risk assessment of heart failure with preserved ejection fraction: A deep learning model	Prospective	CNN	12-lead ECG	First Hospital of Hebei Medical University (China)	238	NR	50.8%	2	No	Prospective internal validation
Raghunath et al.^10^	2020	Prediction of mortality from 12-lead electrocardiogram voltage data using a deep neural network	Retrospective	CNN	12-lead ECG	Geisinger (USA)	1,169,662	58 ± 18	47%	34	No	Internal fivefold cross-validation
Dhingra et al.^11^	2025	Heart failure risk stratification using artificial intelligence applied to electrocardiogram images: a multinational study	Retrospective	CNN	12-lead ECG	YNHHS (USA)	286,880	57	43.4%	10	Yes	UK Biobank (UK) ELSA-Brazil (Brazil)
Lin et al.^12^	2025	A multitask deep learning model utilizing electrocardiograms for major cardiovascular adverse events prediction	Retrospective	CNN	12-lead ECG	CGMH (Taiwan)	2,821,889	60 ± 14.5	50.2%	12	Yes	TSGH (Taiwan)
Butler et al.^13^	2023	A generalizable electrocardiogram-based artificial intelligence model for 10-year heart failure risk prediction	Retrospective	CNN	12-lead ECG	ARIC (USA)	14,613	54.2	44.7%	10	Yes	MESA (USA)
Sun et al.^14^	2023	Towards artificial intelligence-based learning health system for population-level mortality prediction using electrocardiograms	Retrospective	DL	12-lead ECG	Alberta (Canada)	1,605,268	65.80 ± 17.25	56.81%	13	No	NR
Lin et al.^15^	2025	ECG-surv: A deep learning-based model to predict time to 1-year mortality from 12-lead electrocardiogram	Retrospective	CNN	12-lead ECG	CGMH (Taiwan)	4,932,573	60.5 ± 16.8	52.5%	12	Yes	Tri-Service General Hospital (Taiwan)

AI: Artificial Intelligence; ARIC: Atherosclerosis Risk in Communities Study; BIDMC: Beth Israel Deaconess Medical Center; BWH: Brigham and Women’s Hospital; CGMH: Chang Gung Memorial Hospital; CODE: Clinical Outcomes in Digital Electrocardiology; DL: Deep Learning; DNN: Deep Neural Network; ECG: Electrocardiogram; ELSA-Brasil: Brazilian Longitudinal Study of Adult Health; HECIUH: Health Examination Center at Inha University Hospital; HHMU: Hospital of Hebei Medical University; Lead I: Lead I (single-lead electrocardiogram); MESA: Multi-Ethnic Study of Atherosclerosis; MGH: Massachusetts General Hospital; NR: Not reported; SaMi-Trop: Minas Gerais State Health Department – Trypanosoma cruzi (Brazilian cohort of Chagas disease patients); TSGH: Tri-Service General Hospital; UK Biobank: United Kingdom Biobank; YNHHS: Yale New Haven Health System.

Across the included studies, the majority (90.9%) employed CNNs as the primary architecture for ECG signal analysis.^6-13,15,16^ Specifically, CNNs were used either alone or in combination with other methods, such as Long Short-Term Memory (LSTM) layers, Residual Networks (ResNet), or Variational Autoencoders (VAEs). One study utilized broader DL approaches without explicitly using CNN architectures, falling under the category of DNNs or general DL.^14^

Sample sizes varied substantially, ranging from 238 patients to more than 4 million ECGs. Study populations primarily consisted of adult patients from hospital systems associated with large biobanks, with mean ages typically ranging from 54 to 65 years. However, some studies did not provide detailed demographic information.^6^ Regarding follow-up duration, the longest reported period was 34 years,^10^ whereas the shortest was two years.^16^

As shown in Figure 2, most cohorts used to train the AI models were based in the United States of America (USA).^6-12^ Two studies used populations from Taiwan,^13,17^ while one study each included populations from China^10^ and Canada.^14^

**Figure 2:** – Geographic distribution of AI-ECG study cohorts. The map highlights the locations of datasets used for training (blue) and external validation (red) of AI-ECG models. Most training cohorts originated from the USA, while validation datasets were concentrated in the UK, Brazil, Taiwan, and the USA.

Studies that employed external validation also used participants from high-income countries, such as the United Kingdom (UK)^6,9,11^ and the USA.^7,8,13^ Only two studies used a cohort from Brazil for external validation,^6,12^ and two studies included populations from Taiwan.^12,15^

All outcomes relevant to this review are presented in Table 2. Additionally, Figures 3 and 4 display forest plots summarizing the predictive performance of AI-ECG models across the included studies. Figure 3 presents discrimination metrics, including the concordance index (C-index) and the area under the receiver operating characteristic curve (AUC/AUROC), while Figure 4 shows effect measures expressed as hazard ratios (HR) for various cardiovascular outcomes, including 95% confidence intervals when available.

**Table 2:** – Summary of AI-ECG Performance Across Clinical Outcomes

Authors	All-cause mortality	Cardiovascular death	ASCVD	Heart Failure	HFpEF	AMI	Stroke	MACE
Sau et al.^6^	C-index 0.775 (0.773–0.776) Event rate: NR	C-index 0.832 (0.831–0.834) Event rate: NR	C-index 0.696 (0.694–0.698) SEER model C-index 0.547 (0.527–0.567) Event rate: NR	C-index 0.787 (0.785–0.789) Event rate: NR	-	-	-	-
Hughes et al.^7^	-	Stanford (12-lead) AUC 0.832 (0.810–0.854), C-index 0.815 (0.798–0.826); Cedars-Sinai (12-lead) AUC 0,781 (0.767–0.795), C-index 0.777 (0.768–0.783); Columbia (12-lead) AUC 0,825 (0.821–0.829), C-index 0.808 (0.806–0.810) Stanford (1-lead) AUC 0.797 (0.773–0.824), C-index 0.781 (0.760–0.793); Cedars-Sinai (1-lead) AUC 0,778 (0.765–0.792), C-index 0.771 (0.764–0.778); Columbia (1-lead) AUC 0,761 (0.757–0.765), C-index 0.755 (0.753–0.757) Event rate: NR	Stanford (12-lead) AUC 0.668 (0.647–0.687), C-index 0.661 (0.652–0.669); Cedars-Sinai (12-lead) AUC 0,632 (0.591–0.674), C-index 0.635 (0.619–0.652) Event rate: NR	Stanford HR 3.2 (2.9–3.5) Event rate: NR	-	Stanford HR 2.0 (1.7–2.5) Event rate: NR	Stanford HR 1.6 (1.4–1.9) Event rate: NR	-
Al-Alusi et al.^8^	HR 1.47 (1.36–1.60), C-index 0.804 (0.796–0.813) Event rate: 32,7% deaths in the high risk group and 13% in the low risk group	-	-	HR 2.26 (1.90–2.69), C-index 0.804 (0.796–0.813) Event rate: 5.9%	-	HR 1.87 (1.69–2.07), C-index 0.733 (0.725–0.742) Event rate: 14.1%	HR 1.30 (1.18–1.44) Event rate: 6.5%	-
Sau et al.^9^	Male HR 1.22 (1.19–1.25), Female HR 1.17 (1.19–1.31) Event rate: 18.4%	Male HR 1.00 (0.63–1.58), Female HR 1.78 (1.18–2.70) Event rate: 3.7%	-	-	-	-	-	-
Gao et al.^16^	-	-	-	-	ACC: 75% (Cohort A), 71.8% (Cohort B) Event rate: NR	-	-	-
Raghunath et al.^10^	ECG AUC 0.855 Events: 14,207	-	-	-	-	-	-	-
Dhingra et al.^11^	YNHHS HR 1.19 (1.15–1.24) UKB HR 2.13 (1.41–3.24) ELSA HR 3.64 (2.27–5.83) Event rate: YNHHS 7.5%, UKB 0.8%, ELSA 1.7%	-	-	YNHHS HR 6.51 (6.11–6.93), C-index 0.718 (0.697–0.738); UKB HR 18.33 (9.90–33.97), C-index 0.769 (0.670–0.867); ELSA HR 32.06 (15.36–66.92), C-index 0.810 (0.714–0.907) Event rate: YNHHS 4.2%	-	YNHHS HR 1.44 (1.04–2.00) UKB HR 3.16 (1.98–5.02) ELSA HR 3.53 (1.4–8.85) Event rate: YNHHS 0.1%, UKB 0.5%, ELSA 0.4%	UKB HR 2.30 (1.36–3.9) ELSA HR 5.74 (2.59–12.72) Event rate: YNHHS 1.6%, UKB 0.5%, ELSA 0.4%	YNHHS HR 2.10 (2.04–2.17) UKB HR 2.79 (2.17–3.6) ELSA HR 4.04 (2.77–5.89) Event rate: YNHHS 10.4%, UKB 1.8%, ELSA 2.5%
Lin et al.^12^	AUROC 0.89, Sensitivity 0.81, Specificity 0.83, AUROC (External) 0.83 Event rate: 6.3% (3 mo), 8.5% (6 mo), 10.0% (9 mo), 11.4% (1 year)	-	-	AUROC 0.90 Sensitivity 0.83 Specificity 0.81 Event rate: 0.6% (3 mo), 0.8% (6 mo), 1.1% (9 mo), 1.2% (1 year)	-	AUROC 0.85 Sensitivity 0.76 Specificity 0.79 Event rate: 0.3% (3 mo), 0.4% (6 mo), 0.5% (9 mo), 0.5% (1 year)	AUROC 0.76 Sensitivity 0.75 Specificity 0.63 Event rate: 0.3% (3 mo), 0.4% (6 mo), 0.6% (9 mo), 0.7% (1 year)	-
Butler et al.^13^	-	-	-	ARIC AUC 0.76 (0.72–0.83) MESA AUC 0.77 (0.74–0.79) Event rate: 3.6% (MESA), 5.5% (ARIC)	-	-	-	-
Sun et al.^14^	30 days: AUROC 0.843 (0.832–0.858), Brier Score 5.97 (5.83–6.12); 1 year: AUROC 0.812 (0.807–0.81.6), Brier Score 11.44 (11.3-11.6); 5 years: AUROC 0.798 (0.791–0.802), Brier Score 17.52 (17.29–17.83) Event rate: 7.6% (30 days) 17.3%, (1 year) 32.9%, (5 years)	-	-	-	-	-	-	-
Lin et al.^15^	CGMH: C-index 0.860 (0.859–0.861) TSGH: C-index 0.813 (0.807–0.814) Event rate: CGMH 5.9% (3 mo), 7.8% (6 mo), 9.3% (9 mo), 10.5% (1 year); TSGH 6.8% (3 mo), 8.5% (6 mo), 9.5% (9 mo), 10.5% (1 year)	CGMH: C-index 0.891 (0.890–0.893) Event rate: NR	-	-	-	-	-	-

Values are presented as point estimates with 95% confidence intervals in parentheses, unless otherwise specified. CC: accuracy; ARIC: Atherosclerosis Risk in Communities Study; ASCVD: atherosclerotic cardiovascular disease; AUC/AUROC: area under the receiver operating characteristic curve; CGMH: Chang Gung Memorial Hospital; CI: confidence interval; ECG: electrocardiogram; ELSA-Brasil: Brazilian Longitudinal Study of Adult Health; HFpEF: heart failure with preserved ejection fraction; HR: hazard ratio; MACE: major adverse cardiovascular events; MESA: Multi-Ethnic Study of Atherosclerosis; mo: months; NR: not reported; SEER: Surveillance, Epidemiology, and End Results (US database); TSGH: Tri-Service General Hospital; UKB: United Kingdom Biobank; YNHHS: Yale New Haven Health System.

**Figure 3:** – Discrimination metrics (AUC and C-index) for AI-ECG models. Forest plot summarizing the predictive performance of ECG-based AI models across different studies and time horizons. Blue markers represent the C-index (Harrell’s concordance index), and orange markers represent the Area Under the Receiver Operating Characteristic curve (AUC/AUROC). Data are presented as point estimates with 95% Confidence Intervals (95% CI). The dashed vertical line at 0.5 indicates the threshold for non-discriminatory (random) prediction.

**Figure 4:** – Hazard Ratios for clinical outcomes associated with AI-ECG models. Forest plot showing the association between AI-derived risk categories and clinical events (Mortality, Heart Failure, MACE, etc.). Points represent Hazard Ratios (HR), and horizontal bars indicate the 95% Confidence Intervals (95% CI). The dashed vertical line at 1.0 represents the null hypothesis (no difference in risk).

### All-cause mortality

Eight studies reported promising results in predicting all-cause mortality using various AI-ECG models. Lin et al.^12^ demonstrated high accuracy, with an AUROC of 0.89 for 1-year mortality and 0.83 for 1-year all-cause mortality in external validation. Sun et al.^14^ found AUROCs of 0.843 for 30-day mortality, 0.812 for one-year mortality, and 0.798 for 5-year mortality, indicating strong performance across multiple timeframes. Raghunath et al.^10^ reported an AUC of 0.855 using ECG-only data and 0.876 when age and sex were incorporated. In the Surv-ECG study, Lin et al.^15^ achieved a C-index of 0.860 for all-cause mortality. Sau et al.^6^ reported a C-index of 0.775, further supporting the predictive potential of AI-based models for mortality risk.

Sau et al.^9^ identified HR of 1.22 for males and 1.17 for females, suggesting that sex discordance may serve as a novel marker of unrecognized cardiovascular risk. Supporting these findings, Dhingra et al.^11^ reported an HR of 1.19 for all-cause mortality using an AI model applied to ECG images in a multinational study that also demonstrated predictive value for other cardiovascular outcomes, including MI and HF. Al-Alusi et al.^8^ showed that individuals classified as high-risk by the HTN-AI model had a significantly higher 10-year mortality rate (21.0%) compared with the low-risk group (5.4%), underscoring the value of AI in identifying high-risk individuals.

There was marked heterogeneity, with populations ranging from 286,880 to almost five million ECGs and a mean follow-up from 3 to 34 years. While several reports claimed external validation, in most cases, these were replications within the same healthcare system or biobank. Only a minority of studies tested their models in truly independent populations with distinct demographic and clinical characteristics.^6,11^

### Cardiovascular death

The ability of AI-ECG models to predict cardiovascular mortality was also validated. Sau et al.^6^ reported a C-index of 0.832 for cardiovascular death prediction. In Hughes et al.,^7^ the model showed an AUC of 0.83 and a C-index of 0.82 for 5-year cardiovascular death using the full 12-lead ECG, with an AUC of 0.80 when using Lead I alone. Sau et al.^9^ reported a HR of 1.78 for females compared with 1.00 for males among individuals with a normal ECG, reinforcing the role of sex discordance as a potential independent predictor of cardiovascular mortality, particularly in women. Lin et al.,^15^ in the Surv-ECG study, further supported this evidence by reporting a C-index of 0.891 for cardiovascular death prediction.

Studies reporting cardiovascular death also exhibited significant heterogeneity. Populations differed in size and follow-up duration. Event rates were not specified in most studies, and many validations labeled as external were conducted in cohorts derived from the same or closely related healthcare systems, raising concerns about the independence of these validations.

### Atherosclerotic cardiovascular disease

AI-ECG models proved effective in predicting ASCVD. Sau et al.^6^ reported a C-index of 0.696 using 12-lead and Lead I ECGs, integrated with clinical and genetic data from the UK Biobank. In Hughes et al.,^7^ the model achieved an AUC of 0.67 for 12-lead ECG and 0.63 with Lead I. However, prediction of ASCVD events demonstrated wide variation across studies in terms of sample sizes and event definitions (e.g., composite endpoints including MI, stroke, and sudden cardiac death vs. narrower definitions). Regarding validation, most validations occurred within related institutions or datasets, and only Sau et al.^6^ performed truly independent external validation.

### Myocardial infarction

AI-ECG models have demonstrated promising performance in predicting the risk of acute myocardial infarction (AMI). Hughes et al.^7^ reported an AUROC of 0.85 for AMI prediction using the SEER score, along with a HR of 2.0. Similarly, Al-Alusi et al.^8^ reported an HR of 1.87. Dhingra et al.^11^ also found an elevated risk, with an HR of 1.44 for incident AMI. Lin et al.^12^ likewise demonstrated high accuracy, reporting an AUROC of 0.85.

The studies were heterogeneous in design, with differences in event definitions, event rates, and sample sizes ranging from thousands to millions. Regarding validation, most studies conducted validation within the same or closely related biobanks or institutions, and only Dhingra et al.^11^ evaluated their models in geographically distinct cohorts.

### Heart failure

AI-ECG models have demonstrated strong performance in predicting heart failure (HF), although studies varied substantially in methodology. The highest performance was observed in Lin et al.,^12^ who reported an AUROC of 0.90 for HF prediction. Dhingra et al.^11^ found HRs of 6.51 in the YNHHS cohort, 18.33 in the UK Biobank, and 32.06 in the ELSA-Brazil cohort for incident HF, demonstrating substantial prognostic strength across diverse populations. In Sau et al.,^6^ a C-index of 0.787 was achieved using 12-lead and Lead I ECGs, highlighting the model’s reliability.

The AI-ECG model developed by Butler et al.^13^ achieved an AUC of 0.76 in the ARIC cohort and 0.77 in the MESA cohort using ECG data alone, with notable improvement when clinical variables were incorporated. When combined with 12 risk factors, the AI-ECG-Cox model achieved an AUC of 0.82 in ARIC and 0.84 in MESA, demonstrating enhanced predictive value through integration of clinical data. In Hughes et al.,^7^ the model showed 76% sensitivity and 82% specificity for HF prediction, further supporting its diagnostic capabilities. Al-Alusi et al.^8^ reported an HR of 2.26. Finally, Gao et al.^16^ reported 75% accuracy in Cohort A and 71.8% accuracy in Cohort B for heart failure with preserved ejection fraction, with balanced sensitivity (71.7%) and specificity (71.9%).

Most validations were conducted in related datasets; only a small number of studies^6,11^ validated their models in demographically distinct populations.

### Stroke

Lin et al.^12^ demonstrated the potential of AI-ECG models in predicting stroke risk, achieving an AUROC of 0.76. In Hughes et al.,^7^ individuals in the Stanford cohort with the highest SEER scores exhibited a 1.6-fold increased HR for incident stroke, adjusted for age and sex, highlighting the model’s ability to identify individuals at elevated cerebrovascular risk early. Dhingra et al.^11^ reported an HR of 2.30 in the UK Biobank, and Al-Alusi et al.^8^ similarly reported an HR of 2.30.

Studies varied in whether they included only ischemic stroke or all stroke subtypes, which contributed to differences in event rates. Validation was often limited to internal replications or datasets from related institutions, with only Dhingra et al.^11^ conducting truly independent external validation.

### Correlation with traditional scores

As presented in Table 3, Lin et al.^12^ demonstrated superior discrimination compared with the Framingham Risk Score (FRS) across multiple outcomes. Dhingra et al.^11^ showed that their AI-ECG model outperformed the Pooled Cohort Equation for heart failure (PCE-HF). In the YNHHS cohort, the AI-ECG model achieved a C-index of 0.718, compared with 0.601 for the PCE-HF. Sau et al.^6^ reported that their AI-ECG model outperformed the PCE-HF in predicting ASCVD, with a C-index of 0.696 versus 0.547 for the SEER model.

**Table 3:** – Summary of Associations With Traditional Clinical Scores

Authors	Study	Correlation with Traditional Scores
Sau et al.^6^	Artificial intelligence-enabled electrocardiogram for mortality and cardiovascular risk estimation: a model development and validation study	AI-ECG (C-index 0.696) outperformed SEER (C-index 0.547) and showed improved discrimination versus traditional risk-based models
Hughes et al.^7^	A deep learning-based electrocardiogram risk score for long term cardiovascular death and disease	Correlation with PCE (Pearson r = 0.218); NRI: 17.8%; reclassified 16% of patients from low to moderate ASCVD risk (PCE-defined). Improved discrimination for cardiovascular mortality compared with PCE, but lower discrimination for ASCVD outcomes
Al-Alusi et al.^8^	A deep learning digital biomarker to detect hypertension and stratify cardiovascular risk from the electrocardiogram	Comparable discrimination to PCP-HF for HF prediction (C-index 0.804 vs 0.806). Superior discrimination vs PCE for MI (0.733 vs 0.681) and stroke (0.704 vs 0.683)
Sau et al.^9^	Artificial intelligence-enhanced electrocardiography for the identification of a sex-related cardiovascular risk continuum: a retrospective cohort study	NR
Gao et al.^16^	Electrocardiograph analysis for risk assessment of heart failure with preserved ejection fraction: A deep learning model	NR
Raghunath et al.^10^	Prediction of mortality from 12-lead electrocardiogram voltage data using a deep neural network	AI-ECG model (AUC 0.876) outperformed FRS (AUC 0.648) and CCI (AUC 0.816)
Dhingra et al.^11^	Heart failure risk stratification using artificial intelligence applied to electrocardiogram images: a multinational study	YNHHS C-index 0.718 (AI-ECG) vs 0.601 (PCP-HF); improved discrimination and reclassification (NRI 21.9%)
Lin et al.^12^	A multitask deep learning model utilizing electrocardiograms for major cardiovascular adverse events prediction	AI-ECG model demonstrated superior discrimination compared with FRS across outcomes (1-year AUROC: HF 0.90 vs 0.64; MI 0.85 vs 0.69; stroke 0.76 vs 0.61; all-cause mortality 0.89 vs 0.52)
Butler et al.^13^	A generalizable electrocardiogram-based artificial intelligence model for 10-year heart failure risk prediction	AI-ECG model was comparable to FHS-HF (AUC 0.77 vs 0.74), with improved performance when combined with clinical variables (AUC 0.84)
Sun et al.^14^	Towards artificial intelligence-based learning health system for population-level mortality prediction using electrocardiograms	NR
Lin et al.^15^	ECG-surv: A deep learning-based model to predict time to 1-year mortality from 12-lead electrocardiogram	AI-ECG outperformed Framingham-based Cox models for cardiovascular death (C-index 0.891 vs 0.734)

AI: artificial intelligence; ASCVD: atherosclerotic cardiovascular disease; AUC/AUROC: area under the receiver operating characteristic curve; CCI: Charlson Comorbidity Index; C-index: concordance index; ECG: electrocardiogram; FRS: Framingham Risk Score; FRS-HF: Framingham Risk Score for Heart Failure; HF: heart failure; HR: hazard ratio; NRI: net reclassification improvement; NR: not reported; PCE: pooled cohort equations; PCE-HF: pooled cohort equations to prevent heart failure; SEER: Surveillance, Epidemiology, and End Results (US database).

Raghunath et al.^10^ demonstrated superior performance in predicting one-year all-cause mortality, surpassing both the FRS (AUC 0.648) and the Charlson Comorbidity Index (CCI) (AUC 0.816), achieving an AUC of 0.876 using ECG-only data. In Butler et al.,^13^ the FRS for HF achieved an AUC of 0.78 in the ARIC cohort and 0.74 in the MESA cohort. Hughes et al.^7^ found a significant correlation with the PCE-HF, reporting a Net Reclassification Improvement (NRI) of 17.8%, correctly reclassifying 16% of patients initially categorized as low-risk into a moderate-risk category.

### Quality of the studies

The quality of the included studies was assessed using the Newcastle–Ottawa Scale (NOS), as shown in Table 4. The NOS evaluates studies across three domains: selection, comparability, and outcome. Each domain contains specific criteria, with a maximum possible score of 9 points.

**Table 4:** – Quality Assessment of the Studies by the Newcastle-Ottawa Scale

Study	Selection				Comparability		Outcome			Total Quality Score
	Representativeness of Exposed Cohort	Selection of Non-Exposed Cohort	Ascertainment of Exposure	Outcome Not Present at Start	Main Factor	Additional Factor	Assessment of Outcomes	Length of Follow-up	Adequacy of Follow-up	
Sau et al.^6^	*	*	*	*	*	*	*	*	*	9
Hughes et al.^7^	*	*	*	*	*	*	*	*	*	9
Al-Alusi et al.^8^	*	*	*	*	*	*	*	*	*	9
Sau et al.^9^	*	*	*		*	*	*	*	*	8
Gao et al.^16^	*	*	*		*	*	*	*	*	8
Raghunath et al.^10^	*	*	*	*	*	*	*	*	*	9
Dhingra et al.^11^	*	*	*	*	*	*	*	*	*	9
Lin et al.^12^	*	*	*	*	*	*	*	*	*	9
Butler et al.^13^	*	*	*	*	*	*	*	*	*	9
Sun et al.^14^	*		*	*	*	*	*	*	*	8
Lin et al.^15^	*	*	*	*	*	*	*	*	*	9

Eight studies achieved the maximum score of 9, indicating high methodological quality. These studies demonstrated strong cohort selection, appropriate comparability based on key factors, and robust outcome assessment with adequate follow-up periods. Three studies scored 8 points due to missing information regarding comparability factors or the absence of confirmation that the outcome was not present at baseline. However, several studies did not report essential demographic characteristics such as age, sex, or ethnicity – factors that are important for assessing external validity and subgroup fairness. Although these omissions are not fully captured by the NOS, they represent relevant limitations when considering the generalizability of AI model performance across diverse populations. Despite these issues, the overall quality of the studies was high, supporting the reliability of the review’s findings.

### Interpretability Techniques in AI-ECG Models

Among the included studies, interpretability techniques were reported in nine out of eleven articles, as presented in Table 5. Saliency maps were the most frequently applied to highlight ECG waveform regions most relevant to the models predictions (e.g., P wave, PR interval).^8,10,12,14,15^ Other strategies included SHapley Additive exPlanations (SHAP),^13,14^ which provided feature attribution in tree-based or CNN models; neuron ablation and CNN weight heatmaps,^16^ which examined internal model behavior through perturbation; and variational autoencoders (VAEs), waveform averaging, and biological association analyses (GWAS, PheWAS) in Sau et al.^6^ and Sau et al.^9^ to explore biological plausibility and ECG morphology patterns. Two studies did not report the use of any interpretability technique.^7,11^

**Table 5:** – Interpretability Techniques Applied in the Included Studies

Study	Interpretability Techniques	Description
Sau et al.^6^	VAE, waveform averaging, PheWAS, GWAS, Cox models	Linked AI-ECG predictions to biological traits and plausible pathophysiological mechanisms
Hughes et al.^7^	NR	Compared performance with PCE and SEER risk scores, but did not report specific interpretability methods
Al-Alusi et al.^8^	Saliency maps	Used to identify waveform segments predictive of hypertension
Sau et al.^9^	VAE, waveform averaging	Visualized ECG morphology associated with sex-based risk discordance; VAE used for dimensionality reduction
Gao et al.^16^	Neuron ablation, CNN weight heatmaps	Perturbed neurons in CNN layers and visualized associated weights; approach similar to occlusion sensitivity analysis
Raghunath et al.^10^	Saliency maps, expert feedback	Provided visual explanations for normal ECGs flagged by AI; included cardiologist feedback to assess interpretability
Dhingra et al.^11^	NR	Used image-based AI-ECG for risk prediction; did not report specific interpretability methods
Lin et al.^12^	Saliency maps	Identified the P wave and PR interval as key mortality predictors using saliency visualization
Butler et al.^13^	SHAP	Applied SHAP to ECG-derived features for heart failure risk stratification; identified most relevant ECG traits
Sun et al.^14^	SHAP, saliency maps	SHAP used in XGB for feature attribution; Grad-CAM applied to CNN to highlight key ECG segments (PR, QRS, ST-T)
Lin et al.^15^	Saliency maps	Applied visual saliency maps for model interpretation

AI-ECG: Artificial Intelligence-enabled Electrocardiogram; CNN: Convolutional Neural Network; ECG: Electrocardiogram; Grad-CAM: Gradient-weighted Class Activation Mapping; GWAS: Genome-Wide Association Study; NR: Not reported; PCE: Pooled Cohort Equation; PheWAS: Phenome-Wide Association Study; PR: PR interval; QRS: QRS complex; SEER: Surveillance, Epidemiology, and End Results (U.S. database); SHAP: SHapley Additive exPlanations; ST-T: ST segment and T wave; VAE: Variational Autoencoder; XGB: XGBoost.

### Clinical application and prospective use of AI-ECG

Regarding clinical application, most studies explored the potential utility of AI-ECG models for early risk stratification, patient monitoring, or population-level screening; however, these remained retrospective or theoretical. Notably, only Gao et al.^16^conducted a prospective application of their DL model: the algorithm was applied at the time of admission in newly recruited patients with suspected heart failure with preserved ejection fraction (HFpEF), and predictions were compared against invasive left ventricular pressure (LVEDP) measurements. This real-time validation scenario represents the only example of model use in a clinical decision-making context within the included studies. Although it represents the only real-time implementation scenario among the included studies, it does not constitute prospective validation for clinical outcome prediction.

## Discussion

The reviewed studies demonstrate substantial advances in the application of AI to ECG analysis for cardiovascular risk stratification. DL techniques, particularly CNNs, have shown the ability to detect subtle ECG alterations associated with hypertension, ventricular dysfunction, and atrial fibrillation—even in tracings considered normal by specialists. These findings reinforce the clinical potential of AI-ECG for the early detection of asymptomatic or subclinical conditions, with the possibility of transforming clinical practice by enabling the identification of high-risk individuals and the implementation of early interventions from a single examination.

In parallel, other important lines of investigation have explored AI-derived ECG biomarkers, such as electrocardiographic age, which have demonstrated strong associations with mortality and cardiovascular outcomes in large population-based cohorts. For example, previous studies have shown that discrepancies between AI-estimated ECG age and chronological age are linked to increased mortality risk and incident cardiovascular events, including findings from community-based cohorts.^18-20^ These approaches highlight the broader potential of AI-ECG to capture latent physiological and biological aging signals. Although such studies were not included in the present review due to our predefined focus on models directly predicting clinical outcomes from ECG signals alone, they provide important complementary evidence supporting the prognostic value of AI-ECG.

Despite this progress, important methodological limitations undermine direct comparisons across studies. The heterogeneity of AI models employed, the diversity of performance metrics used, and the variation in clinical outcomes analyzed hinder the consolidation of results. A crucial consideration concerns the distinction between statistical validity and clinical validity: although metrics such as AUROC and HR indicate high performance, it remains essential to determine whether the abnormalities identified by the models correspond to physiologically meaningful phenotypes with practical implications for clinical management. Otherwise, there is a risk that algorithms may detect only statistical signatures without genuine clinical relevance.

An analysis of the results shows that, for all-cause mortality, the studies employed different metrics – such as AUROC and HR – as well as varying follow-up periods (e.g., 1-year vs. 5-year mortality), which complicates direct comparisons. Nevertheless, in studies using the same metrics, the models demonstrated consistent performance across all time horizons. A similar limitation was observed for cardiovascular mortality, although all models reported high predictive accuracy. Only two studies evaluated ASCVD, each using distinct metrics, which limited direct comparisons; despite slightly lower discrimination compared with all-cause mortality, results remained satisfactory. For acute myocardial infarction, four studies reported outcomes: three using HR values ranging from 1.44 to 3.53 across different cohorts, and two reporting AUROC values around 0.85, indicating consistent predictive performance. Heart failure was among the outcomes with the highest reported performance, achieving an AUROC of 0.90 and a HR of 32.06. Finally, some studies also assessed stroke, reporting HRs between 1.6 and 2.3, supporting the ability of AI-ECG models to capture risk across multiple cardiovascular outcomes.

AI-ECG models tend to demonstrate stronger performance for all-cause mortality compared with more specific cardiovascular outcomes such as ASCVD. This difference likely reflects the fact that all-cause mortality encompasses a broader spectrum of systemic and cardiac abnormalities that may be detectable in ECG signals, whereas atherosclerotic disease does not consistently produce measurable electrical or structural changes before clinical events occur. As a result, subclinical atherosclerosis may remain undetected by ECG-based models, limiting their predictive performance for ASCVD. Additionally, ASCVD prediction showed substantial heterogeneity across studies, including differences in sample size and outcome definitions, which further restricts direct comparability and may contribute to variability in reported results.

Comparisons between AI-ECG models and traditional risk scores should be interpreted with caution, as many conventional tools were not originally designed to predict outcomes such as all-cause mortality. Differences in intended prediction targets may partially explain variations in reported performance metrics and limit direct comparability. From a clinical perspective, these findings suggest that AI-ECG models and traditional risk scores may serve complementary roles: while conventional tools remain more appropriate for estimating disease-specific risk (e.g., ASCVD), AI-ECG may offer broader risk stratification by capturing global physiological signals, thereby supporting a more integrated assessment of patient risk.

Nonetheless, methodological gaps remain. Not all studies reported the number of clinical events, some omitted calibration metrics, and many focused on short-term outcomes, limiting extrapolation to long-term risk. Another underexplored aspect is temporal generalization: most models were developed in static cohorts, without assessing whether performance remains stable over time in populations subject to epidemiological, therapeutic, or lifestyle changes. Continuous model updating and prospective monitoring will be necessary to ensure lasting clinical utility of AI-ECG.

Additionally, our study selection was restricted to models using ECG signals as the sole input variable, enhancing consistency but not fully reflecting real-world clinical practice, where multiple patient factors are considered. Therefore, when combined with traditional clinical variables, AI-ECG has the potential to function as a digital biomarker, improving the identification of high-risk individuals and reducing underdiagnosis. Future research should advance further by integrating AI-ECG with laboratory data, imaging studies, and even information from wearable devices, moving toward a multimodal approach capable of personalizing cardiovascular risk stratification.

The scalability of AI-ECG represents a major advantage, particularly in resource-limited settings, given the accessibility and simplicity of ECG acquisition. However, most models were developed and validated using data from high-income countries, predominantly the United States, which limits their generalizability. In many cases, validations consisted of replications within related healthcare systems, biobanks, or populations with similar demographic and clinical profiles, rather than assessments in distinct and heterogeneous cohorts. Distinguishing between internal replication and genuine external validation is essential, as the latter provides the most rigorous test of the robustness and transportability of AI-ECG models.

The study by Sau et al.^6^ was among the few to perform external validation in a geographically or demographically distinct population—specifically in Brazil—providing stronger evidence of generalizability. A second study, by Dhingra et al.,^11^ also included a Brazilian cohort, confirming that even in populations with different sociodemographic and clinical characteristics, the models maintained strong performance. Al-Alusi et al.^8^ conducted both internal and external validations in similar populations, with minor differences, such as a higher proportion of women and Black individuals in the external cohort. Nevertheless, comorbidity profiles were largely comparable across groups. Conversely, Gao et al.^16^ did not report detailed population characteristics, limiting cohort comparisons. Similarly, in the studies by Lin et al.^12^ and Lin et al.,^15^ external validation was limited due to the absence of clinical information, allowing only the assessment of one-year mortality as an outcome.

The limited number of truly independent validations, combined with incomplete demographic data, remains a major barrier to the widespread clinical adoption of these tools, as it hampers the evaluation of population diversity and reduces both comparability and interpretability of results.

Among the strengths of the included studies are the use of large datasets, advanced AI methodologies – particularly CNNs – and extended follow-up periods. CNNs demonstrated notable effectiveness in analyzing raw ECG signals, capturing temporal and spatial features with minimal preprocessing. In contrast, studies categorized as DNNs or general deep learning often lacked architectural specificity and provided limited interpretability.

In this context, most of the analyzed studies employed interpretability techniques to enhance the transparency of AI models applied to ECG, reflecting the growing demand for explainable systems. The most commonly used method was saliency maps, which generate heatmaps over the ECG tracing to highlight regions presumed to influence predictions, such as the P wave, QRS complex, or PR interval. Despite their popularity, evidence from medical imaging research indicates that such maps may be unstable, poorly reproducible, and, in some cases, provide misleading explanations that appear plausible but do not reflect the model’s true reasoning.^21,22^

SHAP was also employed in several studies. This method, grounded in game theory, assigns quantitative values to the contribution of each variable to the model’s output, enabling the quantification of each feature’s influence. Unlike purely gradient-based methods, SHAP provides an additive attribution framework that integrates local and global interpretations, offering greater consistency between the two. Its ability to generate intuitive visualizations – such as color plots highlighting important pixels in images or temporal segments in ECG signals – has made it particularly valuable in biomedical applications. Limitations include high computational cost, challenges related to highly correlated temporal variables, and potential inconsistencies in capturing feature dependencies.^23,24^

Other interpretability approaches, including neuron ablation combined with CNN weight heatmaps, VAEs, and biological association analyses (e.g., GWAS, PheWAS), have also been explored to provide additional insights into model behavior. These techniques can shed light on latent representations or the biological plausibility of predictions, but they are often constrained by technical complexity, limited model accessibility, and underlying data biases.^22-24^

Overall, the current landscape indicates that although multiple interpretability techniques are available, their integration into clinical practice remains limited. As observed by Yanagawa and Sato,^22^ future efforts should prioritize methods that not only clarify model predictions but also bring clinical understanding closer to ECG patterns, potentially uncovering subtle physiological markers or disease subtypes not easily identifiable through conventional analysis. Combining interpretability with systematic validation and prospective clinical assessment may transform AI models from purely predictive tools into instruments that actively support clinical reasoning and hypothesis generation.

Notably, only Gao et al.^16^ prospectively tested AI-ECG in a real-world setting, applying the model at patient admission to estimate the risk of HFpEF in comparison with invasive measurements. While models such as AIRE6 and ECG-surv^15^ demonstrated strong associations with physiological characteristics and survival, broader clinical implementation remains incipient.

For AI-ECG to transition from experimental use to clinical practice, integration with healthcare systems and electronic health records is essential. Regulatory frameworks must evolve to evaluate models not only on accuracy but also on fairness, transparency, and clinical utility. Investments in infrastructure, interoperability, and training of healthcare professionals are required to ensure the safe and effective use of these technologies. Prospective clinical trials are equally critical, particularly in primary prevention contexts.

Finally, public health strategies may benefit from AI-ECG by enabling the early detection of high-risk individuals, potentially reducing long-term costs and improving outcomes. However, ethical considerations – such as algorithmic transparency, data privacy, accountability, and equitable access – must be addressed. Models trained on homogeneous, privileged populations risk perpetuating health disparities unless adapted to diverse clinical and demographic contexts.

In summary, the effective use of AI-ECG demands a transdisciplinary approach that bridges technological innovation, clinical practice, public health, and ethics. Only through this lens can AI-ECG evolve from a promising tool into a transformative instrument for reducing health inequities, optimizing care, and advancing population health.

## Conclusion

The application of AI to ECG analysis represents a promising advancement in personalized cardiovascular risk assessment. Nonetheless, further research is needed to ensure the safety, effectiveness, and equitable clinical integration of these technologies. The current evidence, while compelling, underscores the need for more diverse populations in training and validation datasets, improved model interpretability, and prospective clinical trials to fully assess the real-world impact and generalizability of AI-ECG models. Only through rigorous validation and thoughtful implementation can AI-ECG transition from a research frontier to a transformative tool in cardiovascular medicine.
